# Use of DNA Methylation Profiling as a Molecular Classification Tool for Paediatric Central Nervous System Tumours: A Middle‐Income Country Population–Based Study

**DOI:** 10.1111/nan.70041

**Published:** 2025-10-01

**Authors:** Mayara F. Euzébio, Felipe L. T. Silva, Iva L. Hoffmann, Juliana S. Ruas, Larissa Akemi Kido, Dieila G. de Lima, Luciano Queiroz, Izilda A. Cardinalli, Ana Luiza Seidinger, Suelen Nascimento, Helder Tedeschi, Renato D. Puga, Priscila Pini Zenatti Sales, Patricia Y. Jotta, Mariana Maschietto

**Affiliations:** ^1^ Research Center Boldrini Children's Center Campinas São Paulo Brazil; ^2^ Postgraduate Program in Genetics and Molecular Biology, Institute of Biology State University of Campinas (UNICAMP) Campinas São Paulo Brazil; ^3^ Boldrini Children's Centre Campinas São Paulo Brazil

**Keywords:** DNA methylation profiling, middle‐income countries, molecular classification, molecular tumour subtypes, paediatric CNS tumours

## Abstract

Paediatric central nervous system (CNS) tumours are the second most common childhood malignancy and the leading cause of cancer‐related mortality in this age group. Histopathological diagnosis can be challenging, particularly for rare or ambiguous tumours, and may result in misclassification. To evaluate the utility of DNA methylation profiling in a middle‐income country, we performed the Infinium MethylationEPIC BeadChip (Illumina) on tumours from 182 paediatric patients treated at a reference centre for paediatric oncology in Campinas, state of São Paulo, Brazil. Data were analysed using the DKFZ/Heidelberg CNS tumour classifier (v12.8). After excluding control tissue, 163 samples (89.6%) were suitable for classification; 135 (74.2%) achieved a calibrated score ≥ 0.9 and were assigned to a methylation class family. Methylation profiling resulted in a tumour subtype for 88 cases (65.7%) and changed the diagnosis in 28 cases (20.9%), identifying several rare tumour subtypes that were identified solely through methylation analysis, confirming the value of this method in improving diagnostic accuracy. This study highlights the utility of DNA methylation profiling for paediatric CNS tumours in a resource‐limited setting and provides a cohort from an underrepresented middle‐income population to international molecular databases.

## Summary

- This first Brazilian series of paediatric CNS tumours, classified by DNA methylation profiling, expands the representation of middle‐income populations in molecular databases.
- DNA methylation profiling refined or changed the diagnosis in more than 50% of paediatric CNS tumours, demonstrating its utility in resource‐limited settings.
- Discordant cases were largely reclassified by integrated molecular criteria, emphasising the current limitations of histopathology alone.

**Abbreviations**
ACMG: American College of Medical Genetics
AE1/AE3: Cytokeratin AE1/AE3 antibody cocktail
AT/RT: Atypical teratoid/rhabdoid tumour
BSA: Bovine serum albumin
CAAE: Certificado de Apresentação para Apreciação Ética (Ethics Committee reference code, Brazil)
CAPES: Coordenação de Aperfeiçoamento de Pessoal de Nível Superior (Brazilian funding agency)
CEX: Illumina Exome Panel v1.2
CHR: Chromosome
CNA: Copy number alteration
CN: Copy number
CNS: Central nervous system
cIMPACT‐NOW: Consortium to Inform Molecular and Practical Approaches to CNS Tumour Taxonomy
DAB: 3,3′‐Diaminobenzidine
DG8: Droplet Generation Cartridge (Bio‐Rad)
DGONC: Diffuse glioneuronal tumour with oligodendroglioma‐like features and nuclear clusters
DKFZ: Deutsches Krebsforschungszentrum (German Cancer Research Center) Brain Tumour Classifier
DP: Depth of coverage
DRAGEN: Dynamic Read Analysis for GENomics (Illumina Bio‐IT Platform)
ddPCR: Digital Droplet PCR
EPIC: Infinium MethylationEPIC BeadChip array
EMA: Epithelial membrane antigen
FASTQC: FastQC quality control tool for sequencing data
FAM: 6‐Carboxyfluorescein dye (fluorescent probe label)
FAPESP: Fundação de Amparo à Pesquisa do Estado de São Paulo (São Paulo Research Foundation)
FFPE: Formalin‐fixed paraffin‐embedded
FOXR2: Forkhead box R2 (gene/fusion subtype)
GFAP: Glial fibrillary acidic protein
GEO: Gene Expression Omnibus
HICs: High‐income countries
hg38: Human Genome Build 38
IHC: Immunohistochemistry
IDAT: Illumina raw microarray data file format
INI1 (SMARCB1): Integrase interactor 1 protein (SMARCB1 gene product)
InterVar: Variant classification tool following ACMG guidelines
Ki‐67: Proliferation index marker
LNA: Locked nucleic acid
LMICs: Low‐ and middle‐income countries
MAF: Mutation allelic frequency
MUT: Mutant allele
MYB, MYBL1, MYC, MYCN: Genes associated with paediatric CNS tumour subtypes
NEXTSeq: Illumina NextSeq sequencing platform
NTC: Nontemplate control
PFA: Posterior fossa A ependymoma
PLAGL1: Pleomorphic adenoma gene‐like 1 (fusion subtype)
PRONON: Programa Nacional de Apoio à Atenção Oncológica (Brazilian funding program)
QC: Quality control
QKI: Quaking (RNA‐binding protein, fusion partner)
Quantasoft: Bio‐Rad software for ddPCR data analysis
RefSeq: Reference Sequence Database
RNA‐seq: RNA sequencing
S100: S100 calcium‐binding protein
SAME: Hospital Medical Registry Centre
SHH: Sonic Hedgehog pathway subgroup (medulloblastoma)
SMARCB1: SWI/SNF‐related matrix‐associated actin‐dependent regulator of chromatin subfamily B member 1
SPECC1L::NTRK2: Fusion between SPECC1L and NTRK2 genes
STAR‐Fusion: RNA‐seq fusion detection software
t‐SNE: t‐Distributed stochastic neighbour embedding
TBST: Tris‐Buffered Saline with Tween‐20
TSO500: TruSight Oncology 500 sequencing panel
UNICAMP: State University of Campinas
VEP: Variant effect predictor (Ensembl)
VIC: Fluorescent dye (probe label)
WES: Whole‐exome sequencing
WNT: WNT pathway subgroup (medulloblastoma)
WT: Wild type
WHO: World Health Organization
YAP1: Yes‐associated protein 1 (gene/fusion subtype)
ZFTA::RELA: ZFTA–RELA fusion gene

## 1 Introduction

Central nervous system (CNS) tumours are the second most common malignancy and the leading cause of cancer‐related mortality in the paediatric population [1]. There is a global disparity, with around 90% of paediatric cancers occurring in low‐ and middle‐income countries (LMICs) [2]. The 5‐year survival rate reaches 70%–80% in high‐income countries (HICs) [3] but often falls below 30% in LMICs [4]. In Brazil, for example, the 5‐year overall survival for ependymomas ranges from 33% to 60% [5, 6, 7], whereas rates of up to 82% are reported in HICs [8, 9]. This disparity is multifactorial, with contributing factors that include underdiagnosis, delayed presentation and limited access to multidisciplinary neuro‐oncology care [10, 11].

Early and accurate diagnosis is a critical determinant of patient outcomes, but histopathology alone can be challenging, particularly for rare or ambiguous tumours, with misclassification rates of up to 30% even among expert pathologists [12, 13]. The integration of genomic data has become increasingly important, as it enables the reclassification of a significant proportion of tumours into molecular subgroups, already with implications for clinical management and patient outcome [14, 15, 16]. Molecular markers have become central to the World Health Organization (WHO) Classification of CNS Tumours, especially for tumour subtypes that are only recognised by molecular techniques [17], yet access to molecular testing for the detection of mutations, gene fusions and other biomarkers remains limited in most LMICs.

DNA methylation profiling, which reflects both the cell of origin and underlying genetic alterations, has emerged as a powerful tool for CNS and other tumour classification [18, 19, 20, 21, 22]. In paediatric cohorts, molecular classification of CNS tumours confirmed the initial diagnosis in approximately 40%, refined it in 13%–25%, and led to a change in diagnosis in 6%–10% of patients, ultimately leading to a change in clinical management in 5% of the children [14, 22, 23, 24].

The growing gap between molecular insights and clinical practice in LMICs contributes to the disparities in outcomes for paediatric CNS tumours [25], as highlighted by the group that organised the Global Academy Neuro‐Oncology Training Seminar, which addresses challenges faced by LMICs in neuro‐oncology [4]. Given the clinical impact of paediatric CNS tumours and the value of methylation‐based classification for improving diagnostic accuracy, this study aimed to evaluate the molecular classification of paediatric CNS tumours treated at a nonprofit paediatric oncology centre in Brazil, with two main objectives: (1) to contribute to international reference datasets by including a population that is often underrepresented in existing studies and (2) to assess the feasibility and diagnostic utility of methylation profiling as a tool to support histopathology, particularly in light of the limitations faced in routinely applying an extensive panel of specific molecular markers to all patients.

## 2 Materials and Methods

### 2.1 Ethics, Eligibility and Data Collection

Ethical approval was obtained from Boldrini Children's Centre (CAAE: 38849614.2.0000.5376; 44219021.6.0000.5376 and 28386820.7.0000.5376). Participants and/or their legal guardians have signed a written informed consent. Patients were eligible if they had a confirmed diagnosis of a primary brain or spinal cord tumour (at diagnosis or relapse), had an adequate frozen sample in the institutional biobank, and were aged ≤21 years. A paediatric pathologist (IAC) and a neuropathologist (LQ) independently assessed tumour histology and reached a consensus in all tumours. All patients received the standard‐of‐care histopathological diagnosis (tumour histology and immunohistochemistry), which was used for patient management. The tumours were reviewed by the pathology team based on the 2016 WHO classification for this study (based solely on histology or the limited immunohistochemical markers available at our institution). Clinical and demographic information was collected from medical records.

### 2.2 DNA Methylation Experiments

Fresh frozen tumour tissues were used for DNA methylation profiling. The frozen samples were not reviewed by a pathologist, but rather an adjacent fragment was formalin‐fixed paraffin‐embedded (FFPE) and used for diagnostic purposes.

Genomic DNA was extracted using the GenElute Mammalian Genomic DNA Miniprep Kit (Sigma‐Aldrich Co. LLC, St Louis, MO, USA; cat. ID: G1N70‐1KT) and quantified with the Qubit dsDNA BR Assay Kit (Thermo Fisher Scientific, Waltham, MA, USA; cat. ID: Q322853), yielding an average DNA concentration of 100 ng/μL. Samples with 260/280 > 1.7, measured by the BioDrop Duo spectrophotometer (Biochrom Ltd., Waterbeach, Cambridge, UK), were bisulfite converted with the EZ DNA methylation kit (Zymo Research, Irvine, CA, USA; cat. ID: D5002) and hybridised on the 850 K Infinium HD EPIC Methylation Array Bead Chip version 1 (Illumina, San Diego, CA, USA). BeadChip arrays were scanned on the NextSeq 550 (Illumina, San Diego, CA, USA), which generated IDAT files for downstream analysis.

### 2.3 Methylation Classifier, Copy Number and t‐Distributed Stochastic Neighbour Embedding Analysis

IDAT files were evaluated for quality using the Illumina Bead Array Controls Reporter (v1.1) and QC report function from the minfi R package [26]. DNA methylation‐based CNS tumour classification was performed using the random forest DKFZ Brain Tumour Classifier (version 12.8) [27]. Detailed information on the classifier and the molecular classes is available online (https://app.epignostix.com/). Copy number (CN) plots were generated as previously described [22]. In brief, the combined signal intensities of methylated and unmethylated probes were compared to a flat genome reference dataset, and the resulting ratios were plotted across the genome. All CN plots were visually inspected. The normalised *B* values were used as input to the MethylResolver R package 0.1.0 to estimate sample purity [28]. For the t‐distributed stochastic neighbour embedding (t‐SNE) analysis, the R package Rtsne (version 0.11) was used for visualisation and dimensionality reduction by interrogating the 10,000 most variable CpG sites as previously described [18, 27].

### 2.4 Assessment of Methylation Results

Molecular classification was initially based on the calibrated score for the molecular family with a threshold calibrated score ≥ 0.9, similar to other studies [29]. These were further analysed for subclass scores, and those scoring < 0.5 were excluded from subsequent analyses. Samples with a calibrated score < 0.9 were excluded from further analysis.

### 2.5 Whole Exome Sequencing

Exome libraries were prepared using 210 ng of input DNA with the Illumina DNA Prep with Enrichment kit according to the manufacturer's instructions (Illumina, San Diego, CA, USA) and sequenced on the NextSeq500 Illumina platform, generating 150 bp paired‐end reads. Reads were aligned against the human reference genome (hg38) using the Illumina DRAGEN Bio‐IT Platform (version 4.3), which included duplicate removal and base quality score recalibration. Somatic variants were identified using the DRAGEN Somatic Pipeline version 4.3 and annotated with Ensembl VEP (RefSeq version 112) to provide functional impact predictions and known disease associations. Target regions were defined using the Illumina Exome Panel v1.2 (CEX). Variants were filtered to include only those with a total depth of coverage (DP) ≥ 10. Automatic classification of variants according to American College of Medical Genetics (ACMG) guidelines was performed using InterVar, followed by manual verification.

### 2.6 RNA Sequencing

RNA extraction and sequencing were performed as previously described [22]. RNA samples were processed using the Illumina Stranded Total RNA Prep, Ligation with Ribo‐Zero Plus (Illumina, San Diego, CA, USA), employing paired‐end sequencing. The quality of the raw transcriptome data was assessed using FASTQC [30], and STAR‐Fusion [31] was utilised to align and annotate fusion transcripts based on discordant read alignments with default settings. ChimeraViz [32] was employed to visualise fusion genes.

### 2.7 Digital Droplet PCR (ddPCR)

ddPCR was performed using the Qx200 Droplet Digital PCR System (Bio‐Rad, CA, USA). A custom assay (Applied Biosystems, Waltham, MA, USA) incorporating locked nucleic acid (LNA) bases was used to detect the *K27M* mutation at *H3.3A* (H3K27me3) [33]. The reaction mixture contained 1 ng of DNA from tumour tissue, ddPCR SuperMix for probes (Bio‐Rad, CA, USA, cat. ID 186‐3023), 600 nM of forward and reverse primers and 250 nM FAM‐ and 200 nM VIC‐labelled probes in a final volume of 20 μL. The mix was loaded into the DG8 Cartridge (Bio‐Rad, CA, USA, cat. ID: 1864008), followed by the addition of 70 μL of Droplet Generation Oil for Probes (Bio‐Rad, CA, USA, cat. ID: 1863005). Thermal cycling parameters were as follows: initial denaturation at 95 °C for 10 min, 40 cycles of 94 °C for 30 s and 58 °C for 1 min and a final step at 98 °C for 10 min, with a hold at 4 °C. A ramp rate of 2 °C/s was used for all steps. Droplet fluorescence intensities were analysed to distinguish positive and negative events, with thresholds defined using nontemplate controls (NTCs). Data were analysed using Quantasoft (version 1.7, Bio‐Rad). Mutation allelic frequency (MAF) was calculated as the number of mutant‐positive droplets divided by the sum of the mutant‐positive (MUT) and wild‐type (WT) droplets.

### 2.8 Immunohistochemistry

Frozen tissue sections were fixed in 100% cold methanol for 15 min, and then the slides were washed in 1× Tris‐buffered saline with 0.1% Tween‐20 (TBST). Endogenous peroxidase activity was blocked by incubation with a hydrogen peroxide blocking solution for 15 min (EnVision Detection Kit, Dako). After additional TBST washes, nonspecific protein binding was inhibited by incubation with 3%–5% bovine serum albumin (BSA) in TBST for 1 h at room temperature. Sections were subsequently incubated with the primary antibody AbFlex Histone H3K27me3 (Active Motif, cat number 91167) for 1 h at 37 °C, followed by TBST washing and incubation with the secondary antibody (EnVision Detection Kit, Dako) for 1 h at room temperature, followed by TBST washing. Immunoreactivity was visualised using 3,3′‐diaminobenzidine (DAB) as the chromogen, with development monitored until the appearance of a brown precipitate. Counterstaining was performed with haematoxylin, followed by rinsing in tap water for 20 min. Sections were dehydrated through ethanol graded solutions (80%–100%), cleared in xylene and cover‐slipped with a permanent mounting medium for microscopy.

## 3 Results

### 3.1 Enrolment of Patients

We present data from consecutive paediatric patients treated at a specialised oncology centre, covering two periods: 2000–2003 and 2019–2023. The study enrolled 182 patients diagnosed with any type of CNS tumour for which a frozen tumour specimen was submitted to the institutional biobank, per established ethical guidelines. The cohort comprised 46 pilocytic astrocytomas (25.3%), 43 embryonal tumours (23.6%), 22 gangliogliomas (12.1%), 19 ependymomas (10.4%) and 52 other tumour types (28.6%) (Table S1).

### 3.2 The Classifier Predicts With a High Score for the Methylation Family for 82.8% of Central Nervous System Tumours

**FIGURE 1:**
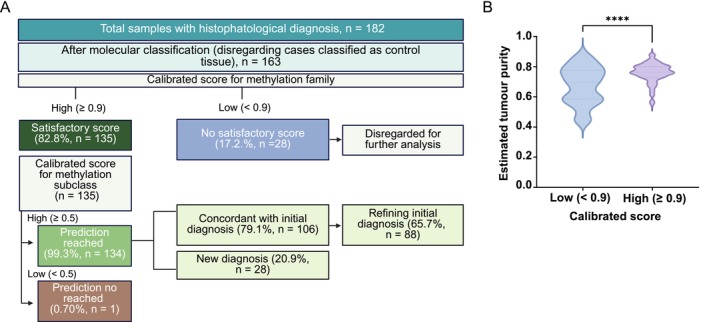
**A)** Overview of the molecular classification of the 182‐paediatric CNS tumours cohort. Excluding cases classified as control tissue (*n* = 19), 135 out of 163 (82.8%) samples were assigned to a methylation family with a high calibrated score of ≥ 0.9. Regarding methylation subclasses, 134 cases (99.3%) achieved a prediction accuracy with a calibrated score of 0.5 or higher. Of these, 106 patients had an initial diagnosis consistent with the molecular classification, while 28 showed discrepancies. In 87 cases, the molecular classification refined the diagnosis by adding a subclass. **B)** Estimated cell purity (percentage) in relation to the calibrated score. **** *p* < 0.0001.

All 182 tumours passed the methylation technical QC criteria and were submitted to the DKFZ brain tumour classifier, version 12.8. Most samples yielded high‐calibrated scores and agreed with the histopathological diagnosis (Figure 1A, Table S2). Notable discrepancies were found among gangliogliomas (32.1%, *n* = 9) and tumours initially diagnosed as CNS embryonal tumours (21.4%, *n* = 6).

Tumour purity was estimated using the MethylResolver R package and subsequently compared to the calibrated score groups (threshold of < 0.90 or ≥ 0.90). Samples with high calibrated scores predominantly exhibited more than 70% of tumour cell content, whereas those with low calibrated scores displayed a reduced tumour cell content (*p* < 0.0001, Figure 1B). Of the 182 samples, 19 were classified as control tissue and were excluded from downstream analysis, pointing to a technical failure in sample provision. From the remaining 163 samples, 135 (82.8%) achieved a calibrated score of ≥ 0.90 for a methylation family. Of these, 134 (99.3%) reached a subclass level calibrated score ≥ 0.5 and were retained for downstream analysis (Figure 1A). Within this subset, methylation profiling refined the initial histopathological diagnosis in 88 tumours (65.7%) and provided a new diagnostic classification for 28 tumours (20.9%) (Table S2). The impact of methylation‐based classification for the 134 tumours is presented below.

### 3.3 The Methylation Classification Refined the Histopathological Diagnosis for Most Tumours

**FIGURE 2:**
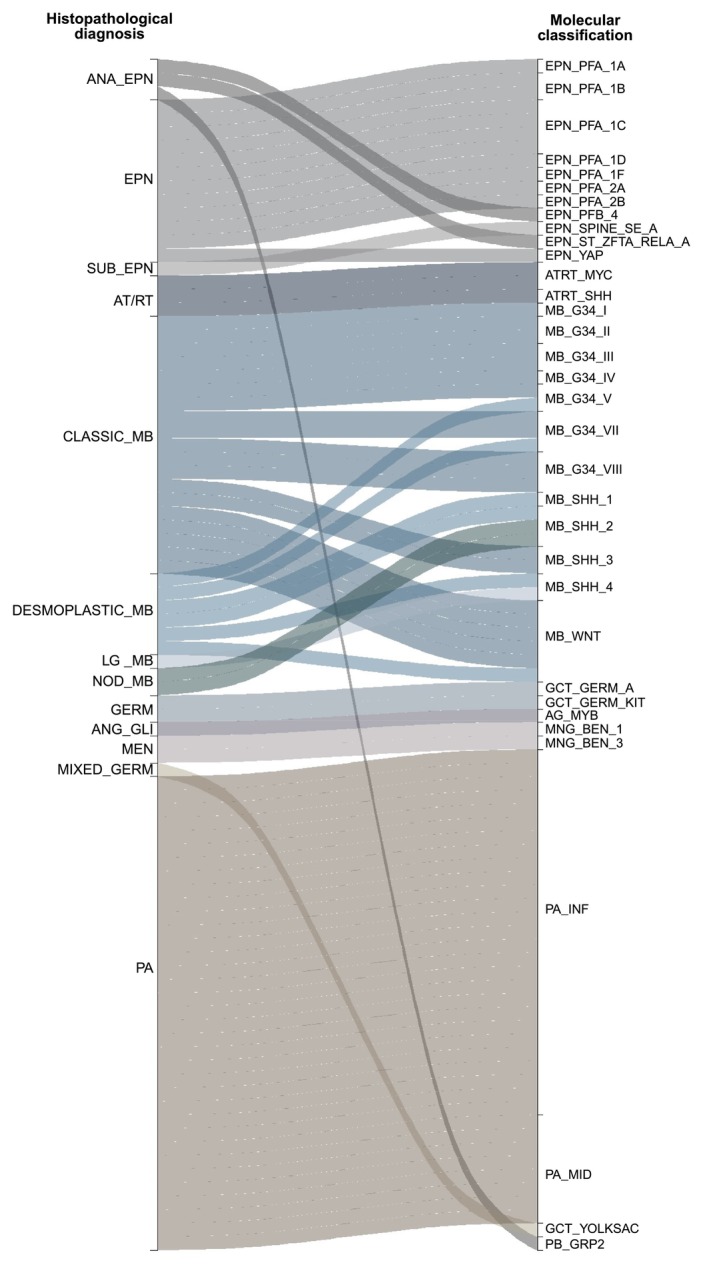
Alluvial plot showing 88 tumours refined by molecular classification (left—initial diagnosis; right—methylation‐based molecular classification). ANA_EPN, anaplastic ependymoma; EPN, ependymoma; SUB_EPN, subependymoma; AT/RT, atypical teratoid rhabdoid tumour; CLASSIC_MB, classic medulloblastoma; DESMOPLASTIC_MB, desmoplastic medulloblastoma; LG_MB, large cells medulloblastoma; NOD_MB, nodular medulloblastoma; GERM, germinoma; ANG_GLI, angiocentric glioma; MEN, meningioma; MIXED_GERM, mixed germ cells tumour; PA, pilocytic astrocytoma; EPN_PFA_1A, Posterior fossa group A (PFA) ependymoma, subclass 1a; EPN_PFA_1B, posterior fossa group A (PFA) ependymoma, subclass 1b; EPN_PFA_1C, posterior fossa group A (PFA) ependymoma, subclass 1c; EPN_PFA_1D, posterior fossa group A (PFA) ependymoma, subclass 1d; EPN_PFA_1F, posterior fossa group A (PFA) ependymoma, subclass 1f; EPN_PFA_2A, posterior fossa group A (PFA) ependymoma, subclass 2a; EPN_PFA_2B, posterior fossa group A (PFA) ependymoma, subclass 2b; EPN_PFB_4, posterior fossa group B ependymoma, subclass 4; EPN_SPINE_SE_A, spinal subependymoma, subtype A; EPN_ST_ZFTA_RELA_A, supratentorial ependymoma, ZFTA fusion‐positive, subtype ZFTA‐RELA fused, subclass A; EPN_YAP, supratentorial ependymoma, YAP1‐fused; ATRT_MYC, atypical teratoid rhabdoid tumour, MYC activated; ATRT_SHH, atypical teratoid rhabdoid tumour, SHH activated; MB_G34_I, Medulloblastoma Group 3, subclass I; MB_G34_II, Medulloblastoma Group 3, subclass II; MB_G34_III, Medulloblastoma Group 3, subclass III; MB_G34_IV, Medulloblastoma Group 3, subclass IV; MB_G34_V, medulloblastoma Group 4, subclass V; MB_G34_VII, medulloblastoma Group 4, subclass VII; MB_G34_VIII, medulloblastoma Group 4, subclass VIII; MB_SHH_1, medulloblastoma, SHH‐activated, subtype 1; MB_SHH_2, medulloblastoma, SHH‐activated, subtype 2; MB_SHH_3, medulloblastoma, SHH‐activated, subtype 3; MB_SHH_4, medulloblastoma, SHH‐activated, subtype 4; MB_WNT, medulloblastoma, WNT activated; GCT_GERM_A, germinoma, subtype A; GCT_GERM_KIT, germinoma, subtype KIT mutant (novel); AG_MYB, angiocentric glioma, MYB/MYBL1‐altered; MNG_BEN_1, meningioma, subclass benign 1; MNG_BEN_3, meningioma, subclass benign 3; PA_INF, infratentorial pilocytic astrocytoma; PA_MID, supratentorial midline pilocytic astrocytoma; GCT_YOLKSAC, yolk sac tumour; PB_GRP2, pineoblastoma, miRNA pathway altered, group 2.

Molecular classification was concordant with histopathological diagnosis in 79.1% of samples (*n* = 106). Among these, 65.7% (*n* = 88) were further classified into a methylation subclass. Pilocytic astrocytoma (35, 39.8%), medulloblastomas (28, 31.8%) and ependymomas (16, 18.2%) were the most frequent tumour types for which methylation classification provided a subclass (Figure 2, Table S2).

For pilocytic astrocytomas, methylation profiling provided a molecular subclass in 35 out of 37 tumours (94.6%), the most common being infratentorial pilocytic astrocytoma (*n* = 27), followed by supratentorial midline pilocytic astrocytoma (*n* = 8). Regarding medulloblastomas, 28 out of 29 (96.6%) tumours agreed with the initial diagnosis and were subclassified into Group 3 and Group 4 (7 tumours each, 25.0%), followed by *SHH*‐activated (8 tumours, 28.6%) and *WNT*‐activated (6 tumours, 21.4%). The molecular subgroups had clinical and molecular characteristics aligned with those described in the literature, including sex distribution, age at diagnosis and outcome (Figure S1). Notably, desmoplastic histology, typically associated with SHH‐activated tumours, was observed in WNT, Group 3 and Group 4 tumours (Figure S2).

For ependymomas, 15 of 16 tumours (93.8%) were subclassified into subclasses, while one tumour had a new diagnosis. The atypical rhabdoid teratoid tumours (AT/RT) were further subclassified into three subtypes: *SHH* (3, 50%), *MYC* (2, 33.3%) and *TYR* (1, 16.7%). Remarkably, 50% of these tumours were not initially diagnosed as AT/RT based on histopathology alone (Figure 2).

**FIGURE 3:**
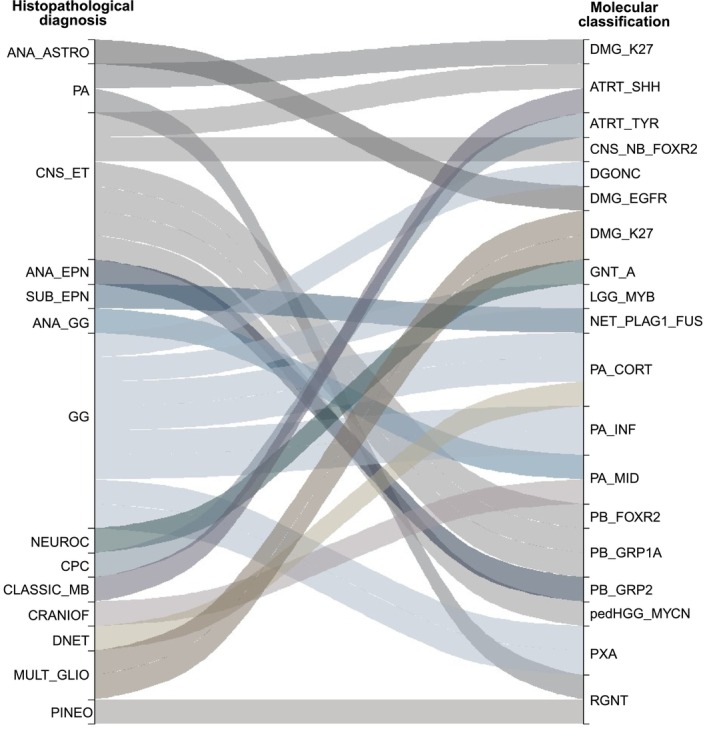
Alluvial plot showing 28 cases that were discrepant with the initial diagnosis and molecular classification (left—initial diagnosis; right—methylation‐based molecular classification). ANA_ASTRO, anaplastic astrocytoma; PA, pilocytic astrocytoma; CNS_ET, CNS embryonal tumour; ANA_EPN, anaplastic ependymoma; SUB_EPN, subependymoma; ANA_GG, anaplastic ganglioglioma; GG, ganglioglioma; NEUROC, central neurocytoma; CPC, choroid plexus carcinoma; CLASSIC_MB, classic medulloblastoma; CRANIOF, craniopharyngioma; DNET, dysembryoplastic neuroepithelial tumour; MULT_GLIO, multiform glioblastoma; PINEO, pineocytoma; DMG_K27, diffuse midline glioma, H3K27‐altered, subtype H3K27‐mutant or EZHIP expressing; ATRT_SHH, atypical teratoid rhabdoid tumour, SHH activated; ATRT_TYR, atypical teratoid rhabdoid tumour, Tyrosinase activated; CNS_NB_FOXR2, CNS neuroblastoma, FOXR2‐altered; DGONC, Diffuse glioneuronal tumour with oligodendroglioma‐like features and nuclear clusters; DMG_EGFR, diffuse midline glioma, H3K27‐altered, subtype EGFR‐altered; GNT_A, Diffuse glioneuronal tumour, subtype A; LGG_MYB_B, diffuse astrocytoma, MYB or MYBL1‐altered, subtype B [infratentorial] (novel); NET_PLAGL1_FUS, neuroepithelial tumour, PLAGL1‐fused; PA_CORT, supratentorial pilocytic astrocytoma; PA_INF, infratentorial pilocytic astrocytoma; PA_MID, supratentorial midline pilocytic astrocytoma; PB_FOXR2, pineoblastoma, MYC/FOXR2‐activated; PB_GRP1A, pineoblastoma, miRNA pathway altered, group 1A; pedHGG_MYCN, diffuse paediatric‐type high‐grade glioma, MYCN subtype; PXA, pleomorphic xanthoastrocytoma; RGNT, rosette‐forming glioneuronal tumour.

Twenty‐eight tumours had molecular classifications that differed from the original histopathological diagnosis (Figure 3), with gangliogliomas and CNS embryonal tumours comprising the majority of discordant tumours (53.6%). Of these, 26 tumours received a molecular subclass that would not have been possible based solely on histopathological diagnosis, except for pleomorphic xanthoastrocytoma.

Rare molecular subclasses were identified in 11 patients, including diffuse astrocytoma, *MYB* or *MYBL1*‐altered; diffuse glioneuronal tumour with oligodendroglioma‐like features and nuclear clusters (DGONC); diffuse glioneuronal tumour, subtype A; diffuse midline glioma, *H3K27*‐altered, subtype *EGFR*‐altered or *H3K27*‐mutant or *EZHIP* expressing; diffuse paediatric‐type high‐grade glioma, *MYCN* subtype; neuroepithelial tumour, *PLAGL1*‐fused; and rosette‐forming glioneuronal tumour. These classifications were verified using orthogonal techniques and CNassessments, as described in the following sections.

### 3.4 Characterisation of CNAs in Newly Diagnosed, Diagnostic Refinement and Rare Molecular Subtypes

**FIGURE 4:**
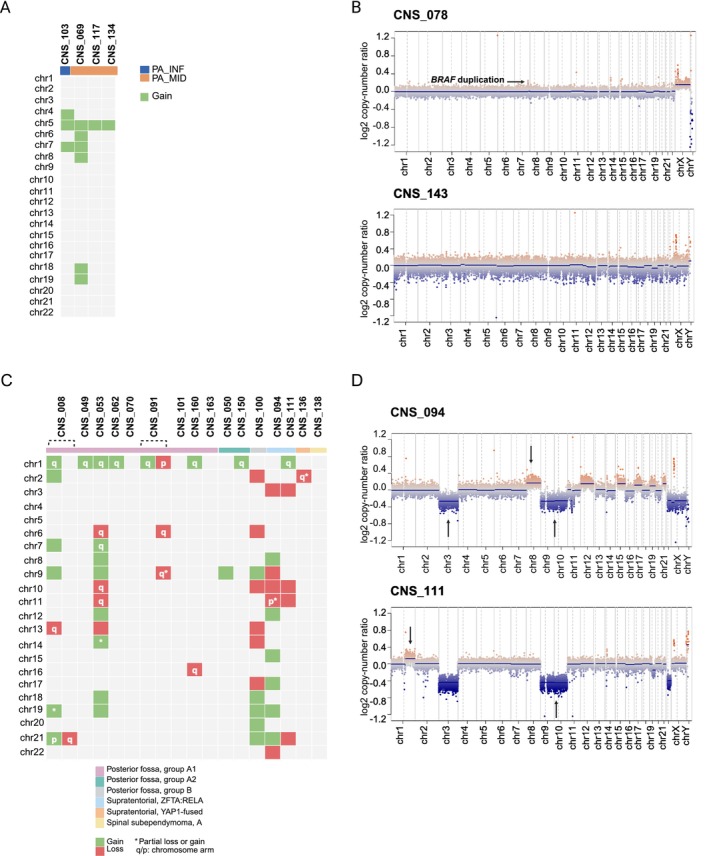
**A)** Oncoplot showing copy number alterations in all pilocytic astrocytomas with nonflat copy number profiling. PA_INF, infratentorial pilocytic astrocytoma; PA_MID, supratentorial midline pilocytic astrocytoma. **B)** Representative copy number of tumours with and without *BRAF* duplication, respectively. Arrow: *BRAF* duplication. **C)** Oncoplot showing the CNAs for 16 ependymomas, grouped according to the molecular subclasses. **D)** Copy number plots of tumours classified as *ZFTA::RELA* fusion‐positive ependymomas. Arrows indicate chromosomal changes usually found in the supratentorial group.

Copy number profiling derived from DNA methylation data enabled the characterisation of tumour group‐specific genomic alterations and the identification of CN associated with specific molecular subclasses. The majority of pilocytic astrocytomas (31 of 35, 88.6%) exhibited a flat CN profile, consistent with the typical low genomic complexity; however, four cases had one or more CNAs, including gains of chr5 (100%), chr 7 and chr11 (50.0% each) as well as chr6 and chr8 (25.0% each) (Figure 4A). Among the 27 infratentorial pilocytic astrocytomas, 25 (92.6%) had *BRAF* duplication, and within the eight supratentorial midline pilocytic astrocytomas, six also harboured *BRAF* duplications (75%), and one tumour showed whole chromosome 7 gain in the absence of focal duplication (Figure 4B).

Ependymomas from the subclass posterior fossa group A (PFA, subgroups A1 and A2) accounted for 68.8% of the ependymomas (11/16) and harboured at least one CNA, with chromosome 1q gain being the most frequent (*n* = 7). CNS_08, CNS_53, CNS_91 and CNS_100 had complex CN profiles, with four or more CNAs. These patients' tumours had aggressive clinical behaviours, with progression and death, despite treatment. CNS_53 and 91 were analysed at recurrence and had concomitantly with chromosomes 1q gain and 6q loss, alterations associated with high‐risk tumours (Figure 4C). Supratentorial *ZFTA::RELA* fusion‐positive ependymomas (CNS_094 and CNS_111) had the typical CNAs, including chromosomes 1q and 8q gain, and chromosomes 3, 9 and 10 loss (Figure 4D).

**FIGURE 5:**
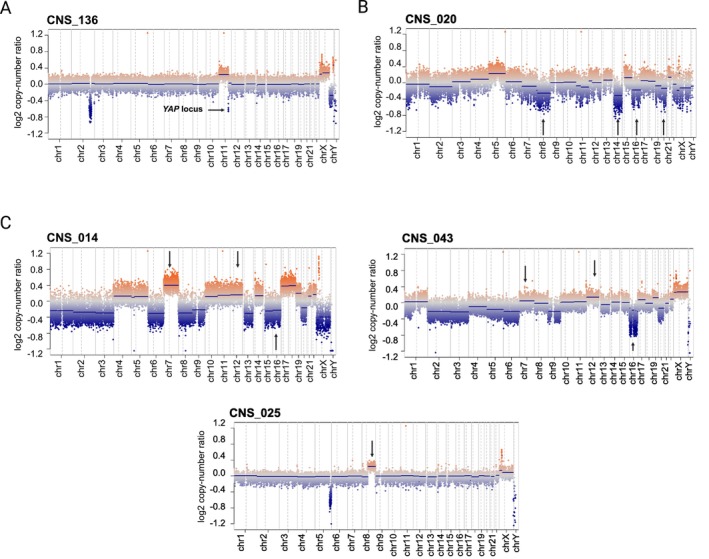
**A)** Copy number plot of a *YAP1*‐fused supratentorial ependymoma showing copy number aberration around the YAP1 locus (arrow), as expected. **B)** Copy number plot of CNS_020, reclassified as pineoblastoma, miRNA pathway altered group 2 subtype, showing several chromosome losses. **C)** Copy number plots of initially diagnosed CNS embryonal tumours. CNS_014 and CNS_043 had chromosome 16 loss and chromosomes 7 and 12 gain, common features of miR pathway altered group 1A subclass; CNS_025 displayed chromosome 8q duplication, typical of *MYC/FOXR2*‐activated subclass.

Reclassified tumours also showed CN patterns congruent with their revised molecular diagnoses. For example, CNS_136, assigned to the *YAP1*‐fused supratentorial ependymoma subclass, had focal loss at the *YAP1* locus, reported in other tumours from the same molecular subtype [34] (Figure 5A) and among the pineoblastomas that received a subclass, CNS_020, assigned to the miRNA pathway altered group 2 subtype, had losses of chromosomes 8, 14, 16 and 20 (Figure 5B) and *DICER1* mutations, NM_177438.3:c.3374G>T and NM_177438.3:c.1849_1850del. Three tumours initially diagnosed as CNS embryonal tumours were reclassified as pineoblastoma, consistent with tumour localisation. CNS_014 and CNS_043 were assigned to the miRNA pathway altered group 1A subtype, with CN profiles showing loss of chromosome 16 and gains of chromosomes 7 and 12 (Figure 5C), which have been described for this molecular class [35]. CNS_025, classified as a pineoblastoma *MYC/FOXR2*‐activated subtype, showed broad 8 chromosomal gain involving the *MYC* locus, similar to other tumours from this molecular subtype [36] (Figure 5C).

Most gangliogliomas were reclassified into rare molecular subclasses. CNS_055 was classified as a diffuse glioneuronal tumour with oligodendroglioma‐like features and nuclear clusters (DGONC), displayed chromosome 14 monosomy and chromosomes 1q and 17q gains (Figure S3A). The histology revealed small cells with round and regular nuclei, dense chromatin and scant cytoplasm, arranged irregularly in neuropil‐like clusters. Giant cells with abundant basophilic cytoplasm and areas with calcified cellular islets were sparsely observed, consistent with DGONC morphology. CNS_115 and CNS_155 were reclassified as pleomorphic xanthoastrocytoma and had the typical *CDKN2A/B* homozygous deletion (Figure S3B).

### 3.5 Characterisation of Samples That Received New Molecular Classes

**FIGURE 6:**
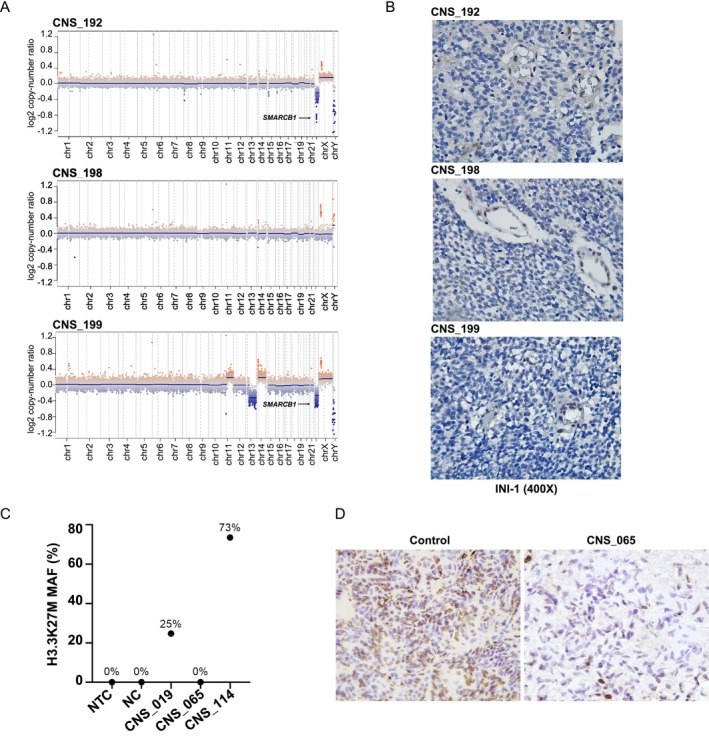
**A)** Copy number plot showing three tumours classified as AT/RT. CNS_192 and CNS_199 showed *SMARCB1* and/or chromosome 21 deletion. **B)** The same three cases presented an absence of INI1 by immunohistochemistry, supporting the new tumour subtype. Tumour vessels were positive for INI1, considered an internal control for the reaction. **C)** *H3K27M* status assessed by ddPCR in the tumour tissue. The scatter plot represents the mutant allele frequency (MAF). Two cases showed high MAF: 25% and 73%, and CNS_065 was not mutated (0%, similar to NC). MAF (%) was calculated as mutant copies per μL/total copies per μL × 100, with the total copies representing the sum of mutant and wild‐type copies. NTC, nontarget control; NC, negative control. **D)** CNS_065 Immunohistochemical staining for H3K27me3 of case CNS_065 shows reduced expression compared to the control sample.

Orthogonal techniques were employed to support molecular classification when suitable material was available, particularly for tumours considered rare molecular subclasses and/or those with diagnoses revised based on methylation profiling. Three tumours changed the original diagnosis to AT/RT: CNS_192 (initially choroid plexus carcinoma), CNS_198 (classic medulloblastoma) and CNS_199 (CNS embryonal tumour). Loss of *SMARCB1* was detected in CNS_192 and CNS_199 (Figure 6A), while all three cases lacked INI1 expression by immunohistochemistry, corroborating the methylation classification (Figure 6B). CNS_192 and CNS_198 received only surgical resection and chemotherapy due to their young age (<18 months). CNS_198 also presented with leptomeningeal dissemination and succumbed to disease progression within 3 months.

In the diffuse midline gliomas *H3K27*‐mutant/*EZHIP* overexpression subtype, the *H3K27* mutation was confirmed in two out of four samples (CNS_019 and CNS_114), whereas CNS_065 tested negative (Figure 6C) and CNS_006 was not evaluated due to a lack of remaining material. Given that *EZHIP* overexpression results in reduced H3K27 trimethylation (similar to the presence of *H3K27A* mutation), immunohistochemical staining for H3K27me3 was performed in CNS_065 and reported reduced nuclear staining compared to the control, thus supporting the methylation‐based classification (Figure 6D).

As expected from cases initially diagnosed based solely on histopathology, and particularly for CNS embryonal tumours, which often lack diagnostic specificity, two additional tumours had their diagnoses reclassified. CNS_156, classified as a diffuse paediatric‐type high‐grade glioma, *MYCN* subtype, had focal *MYCN* amplification (Figure S4A). CNS_104, classified as neuroblastoma with *FOXR2* activation, had a CN profile characterised by gain of chromosomes 1q and 17q, and loss of chromosomes 6q, 10q and 16q, consistent with previously described patterns for this molecular subclass [37] (Figure S4A).

Two tumours were reclassified as rosette‐forming glioneuronal tumours. CNS_016, initially diagnosed as pineocytoma, harboured pathogenic mutations in *FGFR1* and *PIK3CA,* alterations frequently reported in this tumour type. CNS_052, initially diagnosed as a pilocytic astrocytoma, carried mutations in *MSH2*, *FBXW2* and *SMAD6*, although it lacked mutations in genes typically associated with these tumours.

CNS_066, initially diagnosed as ganglioglioma, was reclassified as an astrocytoma with *MYB* or *MYBL1* alterations. RNA sequencing identified the oncogenic fusion *MYB/MYBL1::QKI*, a known driver of this tumour subtype (Figure S4B). CNS_127, originally diagnosed as craniopharyngioma, was classified as a supratentorial midline pilocytic astrocytoma and exhibited a relatively flat genome.

For tumours lacking material for whole exome sequencing (WES) or RNA sequencing, t‐SNE–based clustering was used to visually assess whether the sample was near the reference cases from the methylation‐based subclass it was assigned. For example, CNS_137, initially diagnosed as a subependymoma located in the right frontal lobe, was reassigned to the neuroepithelial tumour, *PLAGL1*‐fused methylation class. Although the CN profiling did not reveal focal alterations involving *PLAGL1*, the tumour had some focal and small CNAs across chromosomes, highlighted in red circles (Figure S4C). Histopathological analysis revealed a low‐grade glial neoplasm composed of astrocytic cells with round, regular nuclei and evenly distributed chromatin, lacking hyperchromatism or atypia and a dense fibrillar structure forming a dense fibrillar network. Immunohistochemistry showed weak GFAP and vimentin expression, and the tumour was negative for cytokeratin (AE1/AE3), S100 and EMA. Ki67 was positive in rare, isolated nuclei (1%). CNS_175, initially diagnosed as a central neurocytoma located at the right foramen of Monro, was classified as a diffuse glioneuronal tumour, subtype A (Figure S4D). The tumour exhibited a relatively flat genomic profile and could not be further evaluated due to insufficient material. Histopathology revealed a low‐grade neuroectodermal neoplasm composed of small, round nuclei and dense chromatin cells and alternating hyper‐ and hypocellular areas with rosette‐like structures. Synaptophysin expression was strong in neutrophil and hypocellular regions, and scattered astrocytic cells were positive for GFAP and vimentin. The tumour had weak S100 expression and was negative for EMA and cytokeratin (AE1/AE3). Rosenthal fibres were observed in the regions adjacent to the tumour. Ki67 labelling index varied from 3% to 5% of tumour cells, with focal hotspots reaching up to 10%.

### 3.6 Tumours Classified Below the Calibrated Score ≤ 0.9

Among tumours with a calibrated score below 0.9 for molecular family (*n* = 18), but ≥ 0.5 for molecular subclass (*n* = 11), five cases were consistent with the initial histopathological diagnosis, while six showed diagnostic discrepancies. For three of these discrepant cases, additional material was available for orthogonal validation (Table S2).

CNS_099, initially diagnosed as pilocytic astrocytoma, was reclassified as adult‐type diffuse high‐grade glioma, IDH‐wildtype, and subtype E. WES sequencing did not identify mutations in *IDH1/2*, corroborating the methylation‐based classification. CNS_173, originally diagnosed as an immature teratoma, was reclassified as a ganglioglioma. This tumour harbours a *PIK3CA* mutation, consistent with the molecular genetic profile frequently associated with gangliogliomas [38]. In addition, t‐SNE clustering placed this sample with other gangliogliomas (Figure S5A). CNS_102, initially diagnosed as desmoplastic ganglioglioma, exhibited an aggressive clinical course inconsistent with the indolent nature of a low‐grade glioma. The methylation profiling reclassified the tumour as pleomorphic xanthoastrocytoma, a class further supported by the presence of a homozygous deletion of *CDKN2A/B* (Figure S5B). Targeted sequencing using the TruSight Oncology 500 (TSO500) panel (Illumina) identified the *SPECC1L::NTRK2* fusion, confirming the molecular class identified by methylation profiling despite the score being < 0.9.

## 4 Discussion

Boldrin Children's Centre is a nonprofit paediatric institution where over 80% of patients receive care through the national public health system. Differing from private hospitals and HIC cohorts, our setting faces significant constraints, including limited access to advanced histopathology and a restricted immunohistochemistry panel, increasing the risk of diagnostic uncertainty, particularly for histologically ambiguous tumours such as CNS embryonal tumours. In the absence of routine access to molecular diagnostics, diagnoses largely rely on conventional histopathology. In this study, frozen tissue was used primarily as a cost‐saving strategy, enabling broader patient inclusion for methylation‐based profiling, which offered valuable insights into the feasibility and potential impact of implementing molecular diagnostics in resource‐limited settings. Even when applied to a selected subset of patients, methylation profiling improves diagnostic precision and supports treatment decisions, with potential socioeconomic benefits [14, 21, 24]. This is particularly critical in the context of paediatric CNS tumours, which often carry poor prognoses and impose long‐term treatment‐related burdens on the developing brain [5, 7, 39].

As a middle‐income country, Brazil's population remains underrepresented in large‐scale genomic and epigenomic studies that contribute to the development of reference databases. While some degree of discrepancy in tumour classification was expected, our findings show that our cohort shares similarities with those reported by high‐income countries, where CNS classification through an agnostic approach has been adopted as a complementary tool [18, 19, 20, 21]. We employed an inclusive strategy by enrolling all cases with frozen tumour tissue stored in the institutional biobank (used for methylation profiling) that achieved the ethical criteria. Despite this broad inclusion approach, our cohort showed a low frequency of high‐grade gliomas, which, according to their epidemiological distribution in the paediatric population, represent 10%–15% of CNS tumours in children [40]. This underrepresentation likely reflects clinical practices at our institution, where these tumours are primarily diagnosed based on imaging and are less frequently biopsied or surgically resected, thereby limiting the availability of tumour tissue for molecular analysis, particularly in a research setting. As a result, most of the analysed samples were low‐grade gliomas (pilocytic astrocytomas and gangliogliomas) and ependymomas, findings that are consistent with previous studies [14, 21, 24].

Across all tumours, our study achieved confident methylation‐based classification in 76.9% of cases. Excluded samples identified as controls—most of which would likely have been excluded with prior pathological assessment—the success rate would increase to 82.8%, comparable to other studies [15, 21, 24]. Samples classified as control tissue highlight the importance of preanalytical evaluation by a pathologist to select appropriate tumour areas before methylation profiling.

Molecular classification refined the diagnosis for 65.7% of the tumours by providing a subclass designation, which would not have been achievable based solely on histology or immunohistochemical markers. This stratification is particularly relevant for medulloblastomas because molecular subgroups are known to have distinct clinical outcomes. Similar to other studies, the WNT‐activated group had a better prognosis, whereas Groups 3 and 4 showed a higher frequency of metastasis and worse outcomes [41, 42]. Therefore, from both prognostic and therapeutic perspectives, the diagnostic refinement offered by molecular classification is especially valuable for medulloblastomas [24, 43, 44].

Similarly, molecular stratification is clinically relevant for patients with ependymomas, as it provides valuable prognostic information. Some molecular subtypes, such as EPN‐PFA and EPN‐ZFTA, are associated with poor prognosis. Additionally, 1q gain and/or 6q loss are associated with a poor prognosis within the EPN‐PFA subgroup [45, 46]. In our cohort, EPN‐PFA exhibited the most complex CN profiles, which included isolated or combined 1q gain and 6q loss, and succumbed to the disease.

Molecular classification changed the initial diagnosis in approximately one‐fifth of cases, with the highest impact on tumours initially classified as CNS embryonal tumours. At our institution, these tumours were historically diagnosed based solely on histopathological features, reflecting the limited availability of ancillary techniques. In our cohort, all tumours originally diagnosed as CNS embryonal tumours were reclassified into well‐defined molecular subtypes, each showing chromosomal alterations consistent with their new classification [47]. These results illustrate the limitations of relying exclusively on histopathology and highlight the value of methylation arrays, especially in LMICs, where specialised neuropathology can be scarce. In such contexts, methylation profiling provides a means to improve diagnostic accuracy, reduce interobserver variability, and enable precise tumour classification. The successful reclassification of CNS embryonal tumours in our study reinforces the value of integrating molecular tools into routine practice, even when applied selectively.

Additional tumours further underscore the value of implementing molecular classification in clinical practice. From a therapeutic standpoint, the change from craniopharyngioma to pilocytic astrocytoma (CNS_127), for example, has relevant clinical implications. Although both tumours are usually managed surgically, pilocytic astrocytomas tend to follow a more favourable clinical course due to their slower growth and lower recurrence rates after resection. Similarly, CNS_055 was initially diagnosed as a low‐grade ganglioglioma but was reassigned to DGONC. This subclass currently lacks driver alterations, making DNA methylation profiling the only method for its identification [48]. Despite histological similarities to other low‐grade gliomas and frequent initial misclassification as high‐grade lesions, DGONC is associated with a relatively favourable prognosis [49].

Some tumours initially classified as high‐grade glioblastomas or pilocytic astrocytomas were reclassified as diffuse midline glioma, *H3K27*‐altered, specifically of *H3K27*‐mutant or EZHIP‐overexpressing subtypes. *H3K27*‐mutant tumours are associated with shorter overall survival compared to the wild‐type tumours. However, recent advances have led to a growing number of experimental therapies and clinical trials targeting this molecular subgroup [50, 51]. These developments underscore the importance of an accurate diagnosis, which can be achieved through DNA methylation profiling.

While a proportion of CNS tumours can be accurately diagnosed using current histopathological criteria, many others benefit from additional molecular characterisation, particularly through DNA methylation profiling, to achieve greater diagnostic precision. DNA methylation classification should be viewed as a complementary diagnostic tool to be integrated with established methodologies. The Consortium to Inform Molecular and Practical Approaches to CNS Tumour Taxonomy (cIMPACT‐NOW) has recently issued recommendations supporting the incorporation of genome‐wide DNA methylation profiling into CNS tumour diagnostics, emphasising the need to integrate this approach with clinical, radiological, histological and genomic data [52]. Despite the use of comprehensive diagnostic tools, a subset of cases remains unresolved. In our study, 25.8% of tumours could not be conclusively classified: 19 samples were categorised as control tissue, and 28 had low calibrated scores. The identification of rare and extremely rare tumours underscores the importance of analysing complete and consecutive samples, which not only help replicate and validate existing findings but also capture the biological and clinical heterogeneity of CNS tumours, especially across diverse populations.

This study demonstrates the feasibility of implementing molecular classification of paediatric CNS tumours in a reference Brazilian centre. We hypothesised that methylation profiling would refine diagnoses in an LMIC setting and reveal discrepancies with potential clinical implications compared with standard histopathology. By presenting a substantial cohort from a middle‐income country, this work addresses the under‐representation of LMICs in international molecular databases and illustrates the practicality of applying methylation profiling in resource‐limited environments. Although associated with higher upfront costs, accurate molecular classification can guide treatment decisions in centres without comprehensive molecular testing. In the long term, enhanced diagnostic precision has the potential to improve outcomes, increase years of life saved, and ultimately yield favourable socioeconomic returns.

## Ethics Statement

The study was conducted following the Declaration of Helsinki and approved by the Institutional Ethics Committee of Boldrini Children's Centre (CAAE: 51389921.8.0000.5376, CAAE: 28386820.7.0000.5376, CAAE: 44219021.6.0000.5376). Informed consent was obtained from all subjects involved in the study.

## Conflicts of Interest

The authors declare no conflicts of interest.

## Supporting information


**Figure S1:**Clinical data and copy number features of medulloblastoma. All WNT‐activated tumours presented monosomy of chromosome 6. SHH‐activated tumours showed loss of 9q and *MYCN* amplification. Only typical CN alterations are reported; the number of cases with each alteration is shown in parentheses. T surgery; *sepsis.

**Figure S2:** nan70041‐sup‐0002‐figure_S2.tif. **A‐B)** Desmoplastic medulloblastoma with nodular architecture and areas of desmoplasia. **D–E, M–N)** Desmoplastic medulloblastoma exhibiting areas of desmoplasia. **G–H, J–K**) Desmoplastic medulloblastoma with a nodular pattern. **P–Q)** Desmoplastic medulloblastoma displaying vaguely nodular areas. **C, F, I, L, O)** Reticulin staining, where reticulin permeates the internodular tissue. Magnification: 200× (A, C, D, F, G, I, J, L, M, O, P, R), 400× (B, E, H, K, N, Q).

**Figure S3:** nan70041‐sup‐0003‐figure_S3.pdf. **A)** Copy number plot showing CNS_055 with monosomy of chromosome 14, gain of chromosomes 1q and 17q (arrows), common features in diffuse glioneuronal tumours. **B)** Copy number plots of two pleomorphic xanthoastrocytomas showing *CDKN2A/B* deletion (left panel) and detailed wrap plots for focal deletion (right panel).

**Figure S4:** nan70041‐sup‐0004‐figure_S4.pdf. **A)** Copy number plot for case CNS_104, displaying chromosomes 1q and 17q gains and chromosomes 6q, 10q and 16q losses, which are some common features of neuroblastoma, *FOXR2* activated subclass; case CNS_156, displayed *MYCN* amplification, a common feature for a high‐grade glioma *MYCN* subclass. **B)** CNS_066 was reclassified as diffuse astrocytoma*, MYB* or *MYBL1*‐altered, displaying oncogenic driver *MYB/MYBL1::QKI* as expected. Circus plot showing all chromosomes containing cytoband information. Links between chromosomal locations (accompanied by gene names) represent fusion events; intrachromosomal and interchromosomal fusions are denoted by red and blue lines, respectively. The number of readings supporting the fusion event determines the breadth of each line. **C)** CNS_137 copy number profile (top panel) showing no alterations at *PLAGL1*, presenting many small CNAs throughout the genome (red circles). t‐SNE (bottom panel) highlighting the sample grouped with neuroepithelial tumour, *PLAGL1*‐fused subgroup. EPN_SPINE_SE_A, spinal subependymoma, subtype A; LGG_MYB_C, diffuse astrocytoma, *MYB* or *MYBL1*‐altered, subtype C [isomorphic]; NET_PLAG1_FUS, neuroepithelial tumour, *PLAGL1*‐fused; EPN_ST_ZFTA_FUS_C, supratentorial ependymoma, *ZFTA* fusion‐positive, subclass C. D) t‐SNE highlighting CNS_175 grouped with diffuse glioneuronal tumour, subtype A subgroup. GG, ganglioglioma; PA_CORT, supratentorial pilocytic astrocytoma; GNT_A, diffuse glioneuronal tumour, subtype A; PGNT, papillary glioneuronal tumour. The colour code represents the methylation subclasses within the reference cohort, providing a visual distinction for readers to assess the relative location of a sample within the t‐SNE, as referenced by the DKFZ Brain Tumour Classifier (version 12.8).

**Figure S5:** nan70041‐sup‐0005‐figure_S5.pdf. **A)** t‐SNE highlighting CNS_173 clustered with the ganglioglioma subgroup. GG, ganglioglioma; GNT, diffuse glioneuronal tumour; PA_INF, infratentorial pilocytic astrocytoma; PA_CORT, supratentorial pilocytic astrocytoma. **B)** Copy number and detailed wrap plots of CNS_102 showing *CDKN2A/B* deletion. The colour code represents the methylation subclasses within the reference cohort, providing a visual distinction for readers to assess the relative location of a sample within the t‐SNE, as referenced by the DKFZ Brain Tumour Classifier (version 12.8).

**Table S1:** Distribution of tumour histology according to the initial diagnosis.

**Table S2:** Supporting Information.

## Data Availability

Methylation EPIC array raw data (idat files) are available at GEO, under accession number GSE296487. The DKFZ group provided the code for running the Brain Tumor Classifier (version 12.8) (https://app.epignostix.com/).
