# Supplementary material for: Use of DNA Methylation Profiling as a Molecular Classification Tool for Paediatric Central Nervous System Tumours: A Middle‐Income Country Population–Based Study
Source: Neuropathol Appl Neurobiol. 2025 Oct 1;51(5):e70041. doi: 10.1111/nan.70041 (PMC12488389; doi:10.1111/nan.70041)
Supplement: Supplementary file 1 — Figure S1:Clinical data and copy number features of medulloblastoma. All WNT‐activated tumours presented monosomy of chromosome 6. SHH‐activated tumours showed loss of 9q and MYCN amplification. Only typical CN alterations are reported; the number of cases with each alteration is shown in parentheses. T surgery; *sepsis. [file NAN-51-e70041-s003.pdf]

Molecular classification	Group 3	Group 4	WNT-activated	SHH-activated
Number of cases	7	7	6	8
**Clinical data**				
Sex (Male / Female)	Male 100%	Male 71.4%, Female 28.6%	Male 50%, Female 50%	Male 37.5%, Female 62.5%
Age (years)	>3–10 (n = 7)	>3–10 (n = 7)	>3–10 (n = 4)<br>>10–17 (n = 2)	0–3 (n = 3)<br>>3–10 (n = 2)<br>>10–17 (n = 3)
Histology	Classic: 6<br>Desmoplastic: 1	Classic: 6<br>Desmoplastic: 1	Classic: 5<br>Desmoplastic: 1	Classic: 2<br>Desmoplastic: 3<br>Nodular: 2<br>Large cells: 1
Metastasis at diagnosis	2	3	2	-
Death by disease (1-10y)	3	2	2	2
Death from other causes	1*	2*	1[T]	1*
**Copy number**				
Broad	7- 10- 14- (1)<br>8q (1)	8- (3)	6- (6)	9p+ 9q- (2)
Focal	*RB1* loss (2)<br>*MYC* gain/amplification (3)<br>*MYCN* gain (2)<br>*TP53* loss (3)	*RB1* loss (1)<br>*TP53* loss (5)		*MYC* gain/ amplification (2)<br>*TP53* loss (1)
