# Supplementary figures and images for: Use of DNA Methylation Profiling as a Molecular Classification Tool for Paediatric Central Nervous System Tumours: A Middle‐Income Country Population–Based Study
Source: Neuropathol Appl Neurobiol. 2025 Oct 1;51(5):e70041. doi: 10.1111/nan.70041 (PMC12488389; doi:10.1111/nan.70041)

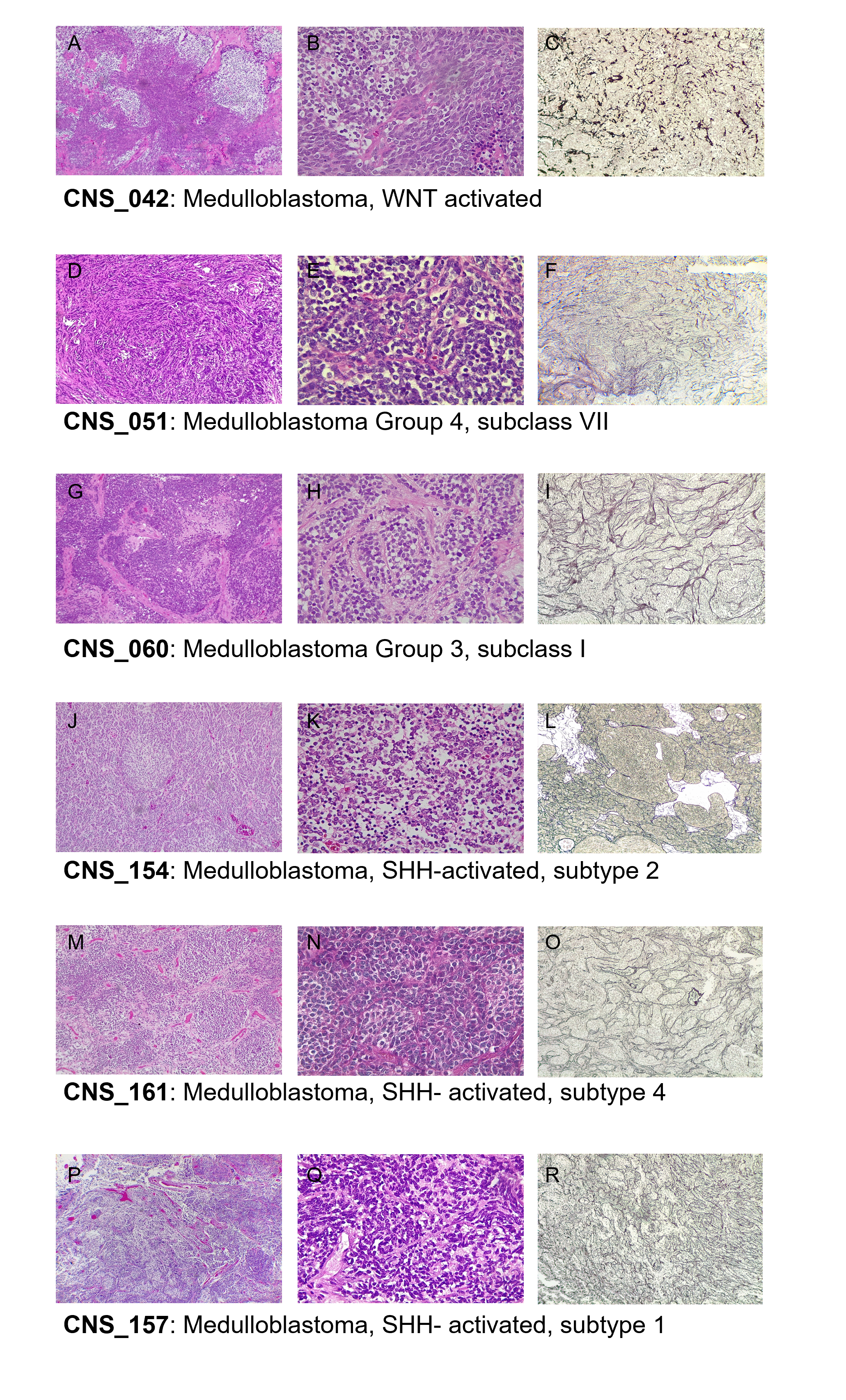

Supplement: Supplementary file 2 — Figure S2: nan70041‐sup‐0002‐figure_S2.tif. A‐B) Desmoplastic medulloblastoma with nodular architecture and areas of desmoplasia. D–E, M–N) Desmoplastic medulloblastoma exhibiting areas of desmoplasia. G–H, J–K) Desmoplastic medulloblastoma with a nodular pattern. P–Q) Desmoplastic medulloblastoma displaying vaguely nodular areas. C, F, I, L, O) Reticulin staining, where reticulin permeates the internodular tissue. Magnification: 200× (A, C, D, F, G, I, J, L, M, O, P, R), 400× (B, E, H, K, N, Q). [file NAN-51-e70041-s004.tif]

A

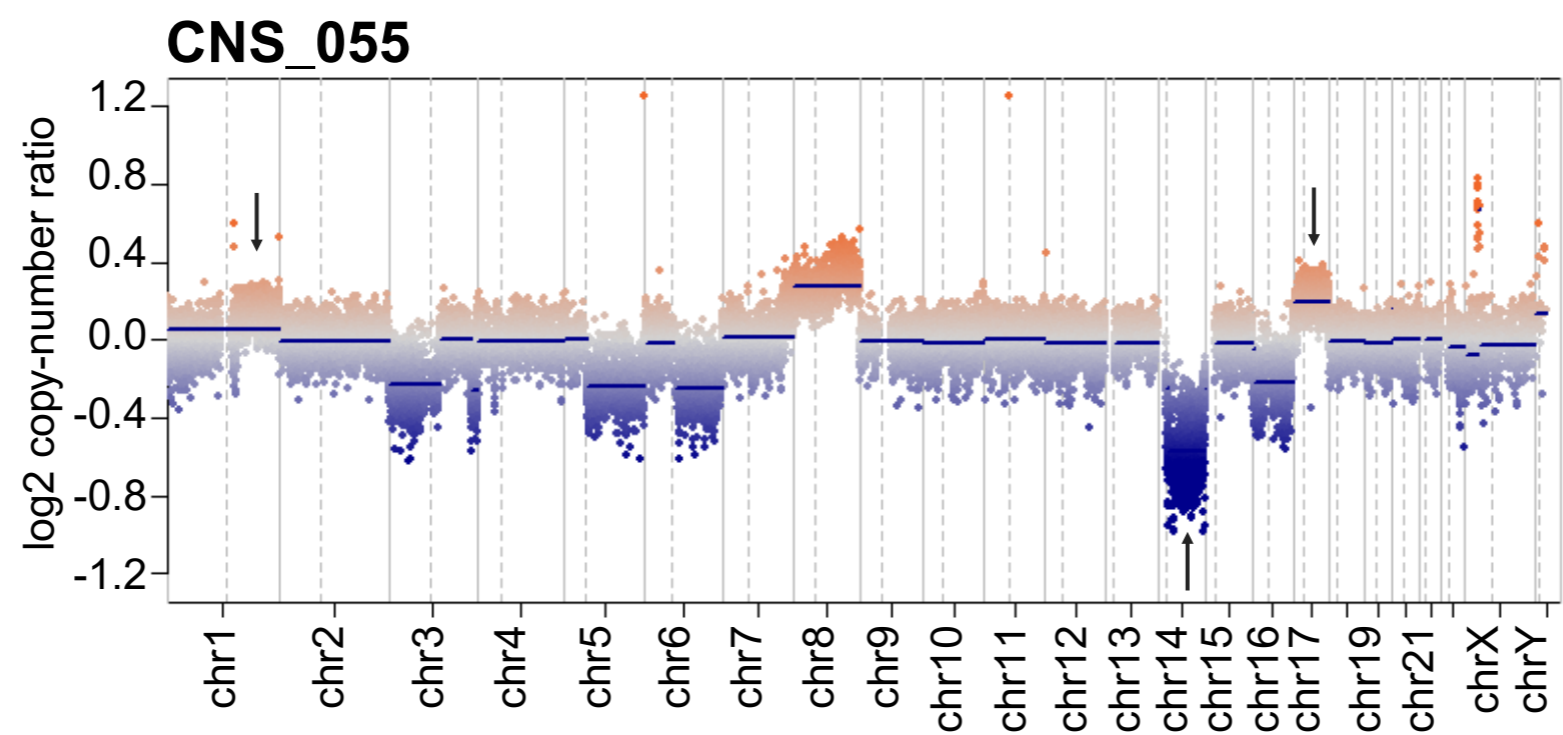

B

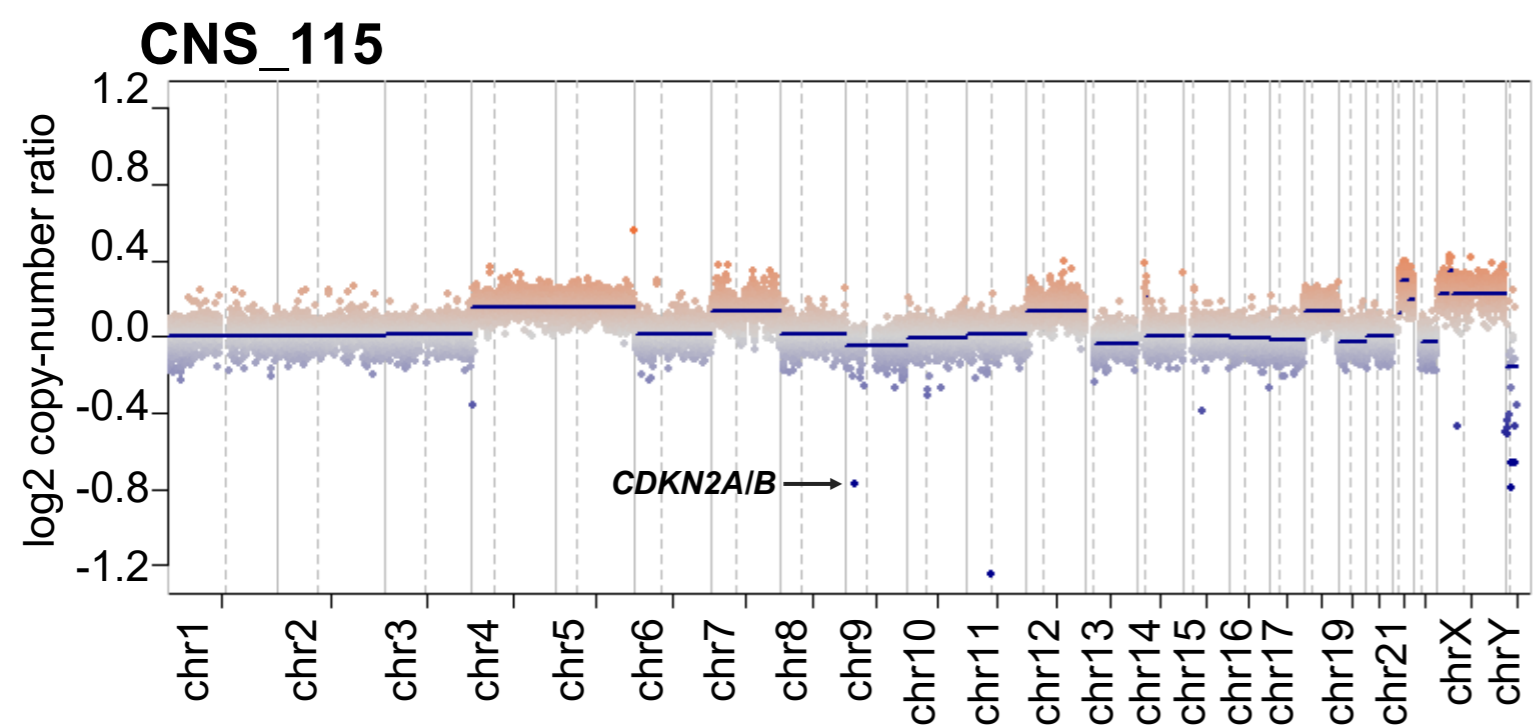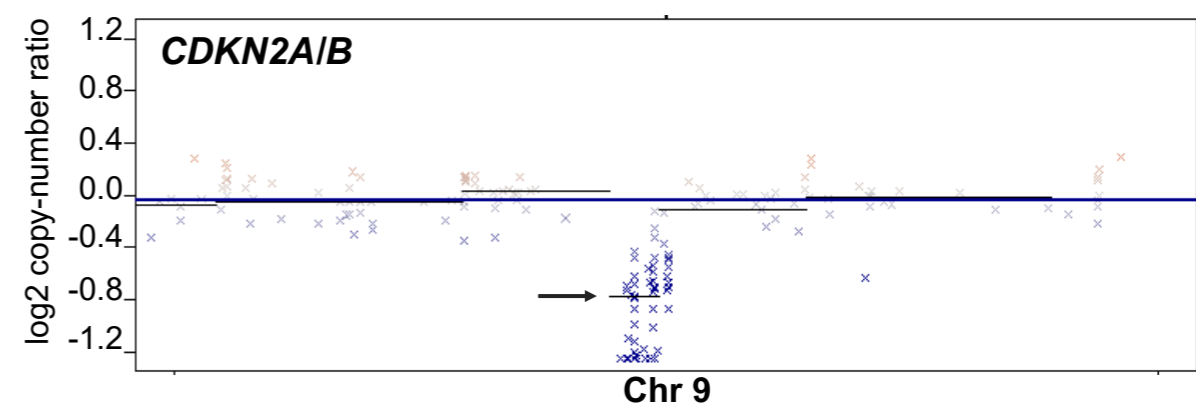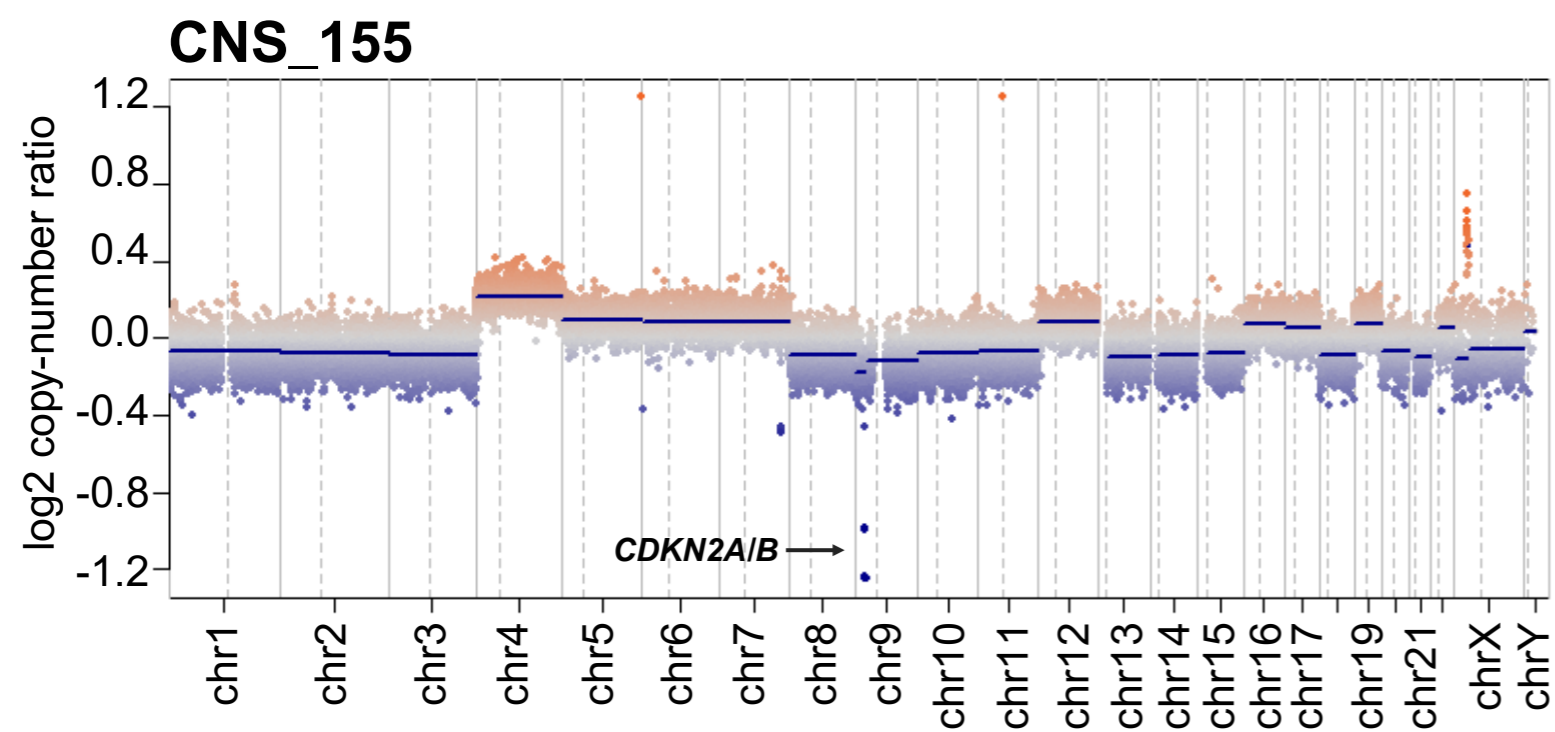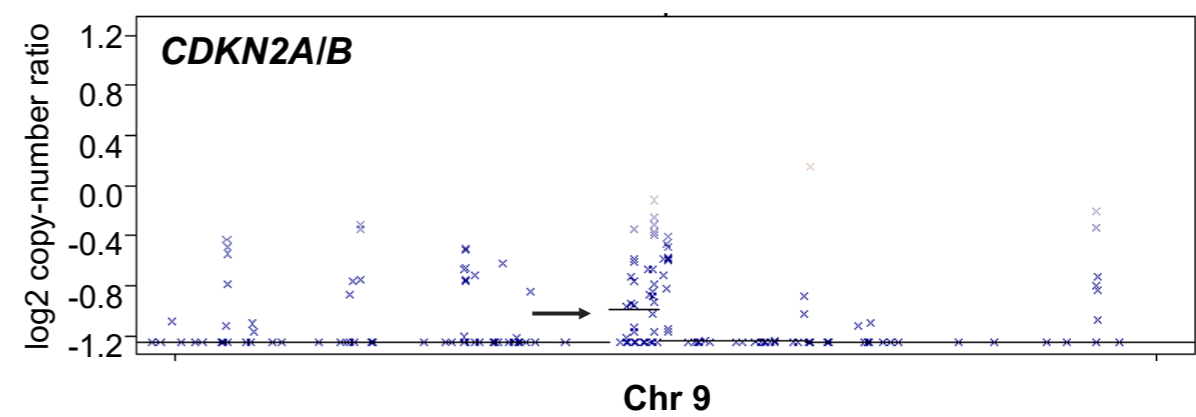

Supplement: Supplementary file 3 — Figure S3: nan70041‐sup‐0003‐figure_S3.pdf. A) Copy number plot showing CNS_055 with monosomy of chromosome 14, gain of chromosomes 1q and 17q (arrows), common features in diffuse glioneuronal tumours. B) Copy number plots of two pleomorphic xanthoastrocytomas showing CDKN2A/B deletion (left panel) and detailed wrap plots for focal deletion (right panel). [file NAN-51-e70041-s006.pdf]

A

## CNS\_104

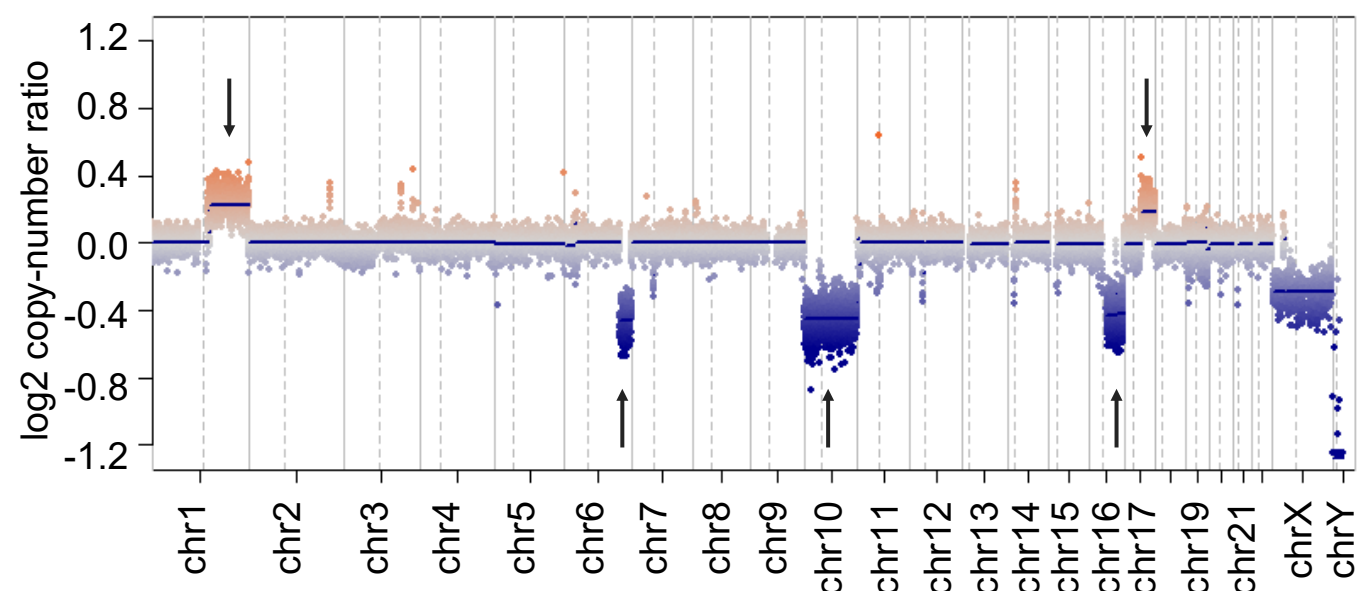

## CNS\_156

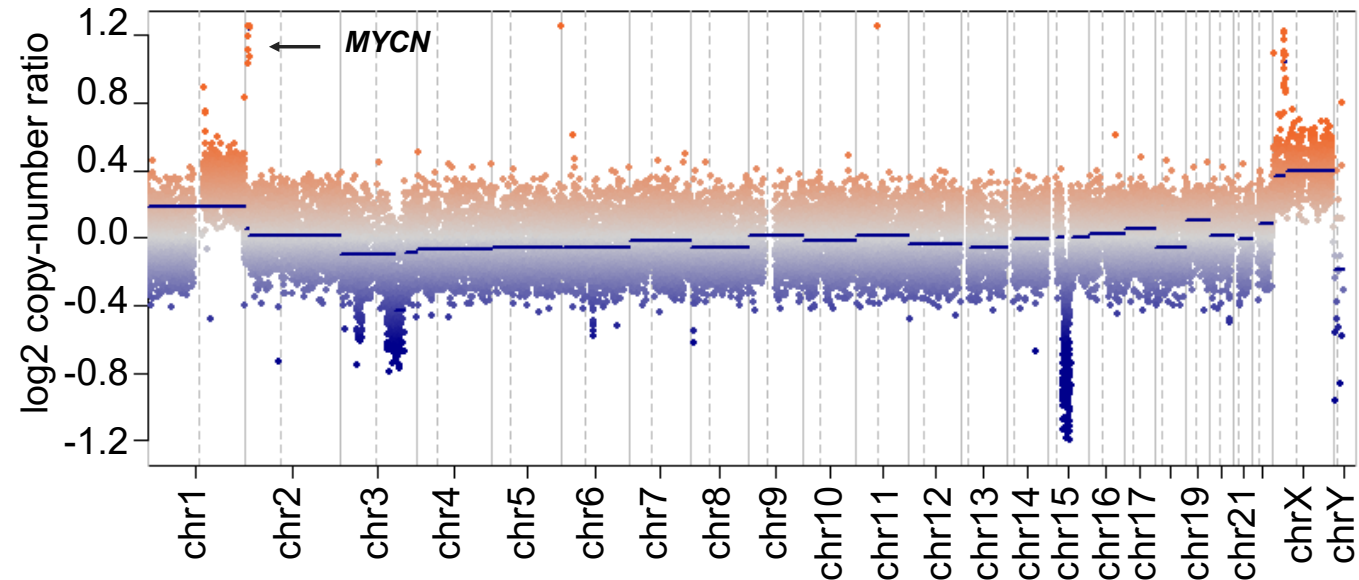

C

## CNS\_137

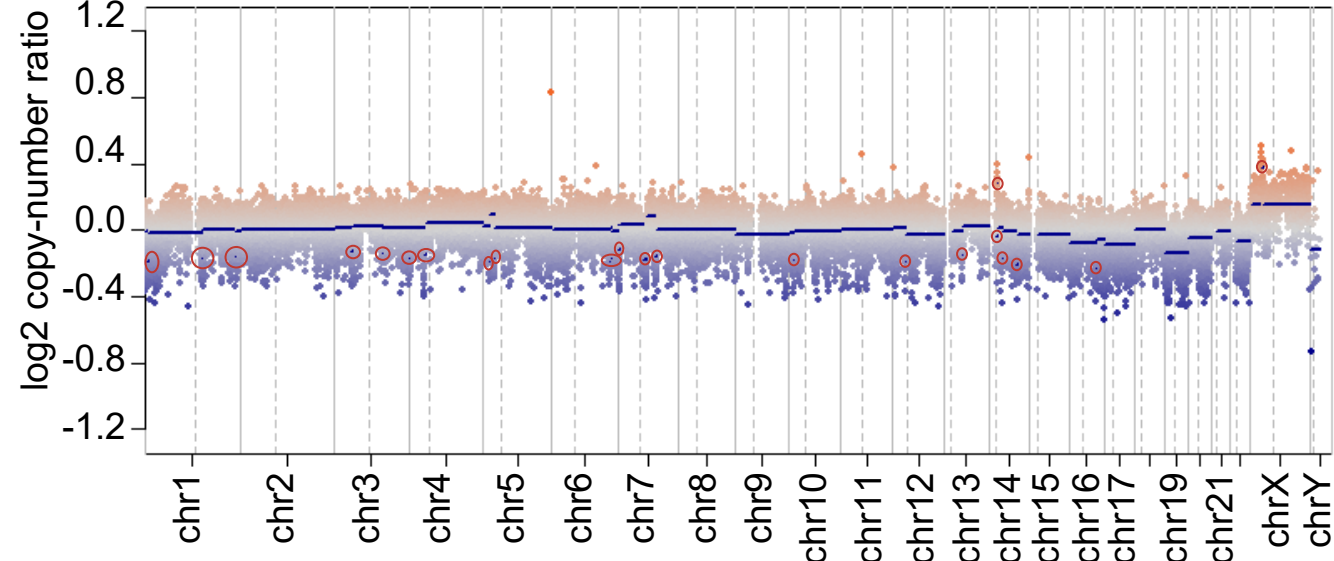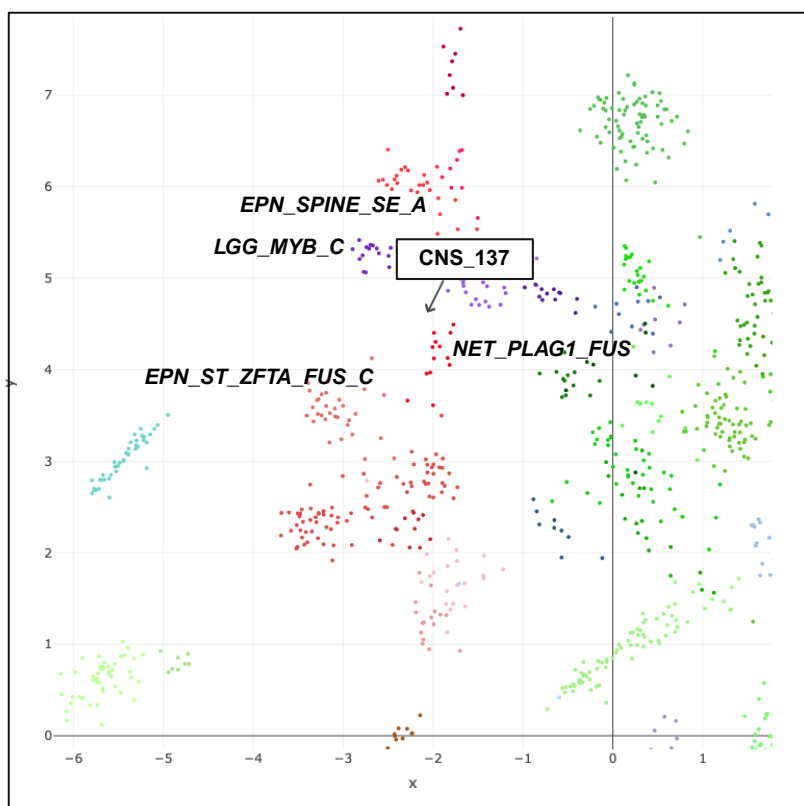

B

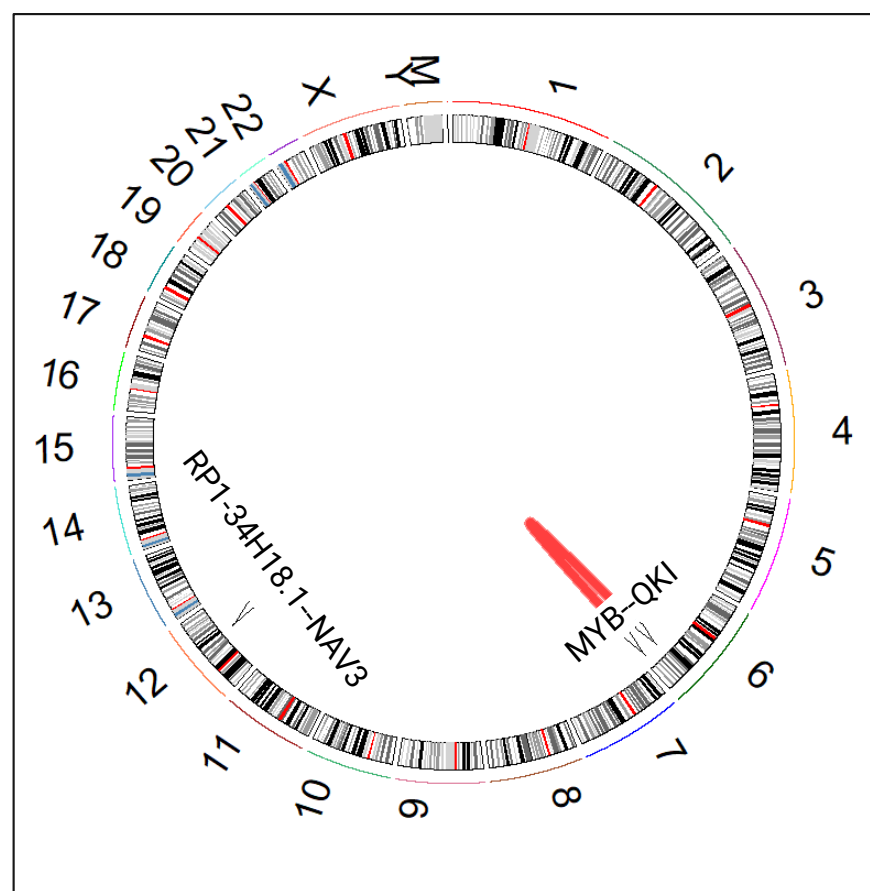

D

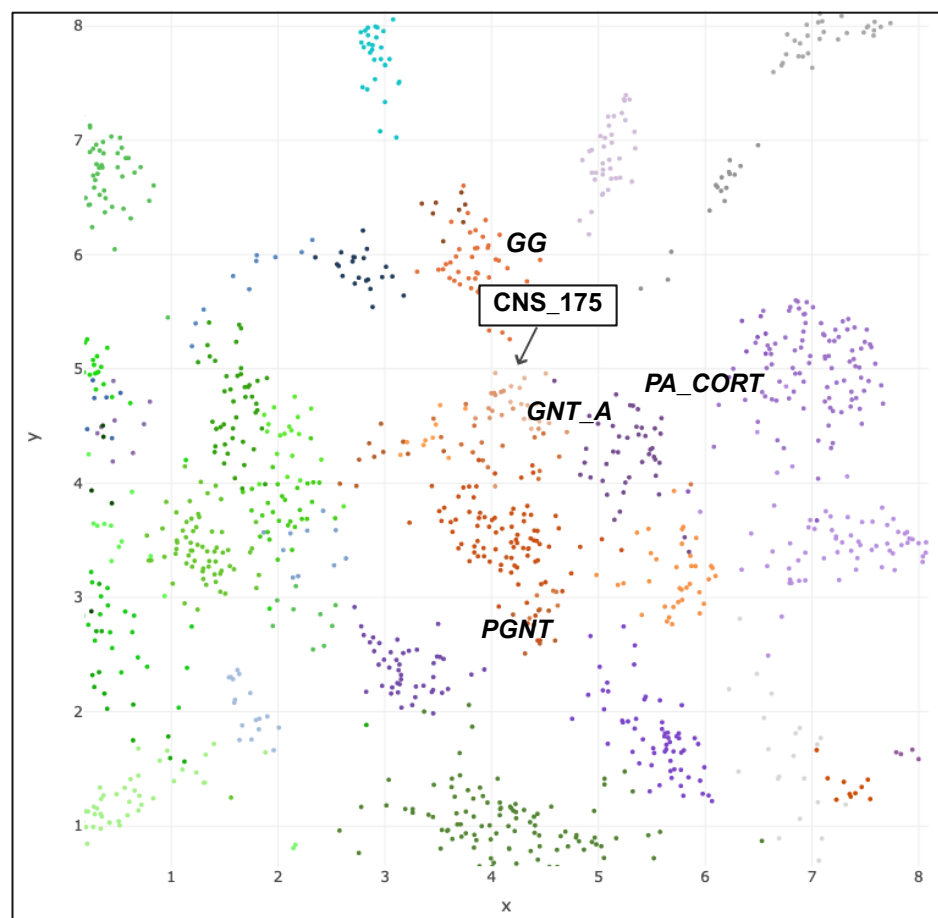

Supplement: Supplementary file 4 — Figure S4: nan70041‐sup‐0004‐figure_S4.pdf. A) Copy number plot for case CNS_104, displaying chromosomes 1q and 17q gains and chromosomes 6q, 10q and 16q losses, which are some common features of neuroblastoma, FOXR2 activated subclass; case CNS_156, displayed MYCN amplification, a common feature for a high‐grade glioma MYCN subclass. B) CNS_066 was reclassified as diffuse astrocytoma, MYB or MYBL1‐altered, displaying oncogenic driver MYB/MYBL1::QKI as expected. Circus plot showing all chromosomes containing cytoband information. Links between chromosomal locations (accompanied by gene names) represent fusion events; intrachromosomal and interchromosomal fusions are denoted by red and blue lines, respectively. The number of readings supporting the fusion event determines the breadth of each line. C) CNS_137 copy number profile (top panel) showing no alterations at PLAGL1, presenting many small CNAs throughout the genome (red circles). t‐SNE (bottom panel) highlighting the sample grouped with neuroepithelial tumour, PLAGL1‐fused subgroup. EPN_SPINE_SE_A, spinal subependymoma, subtype A; LGG_MYB_C, diffuse astrocytoma, MYB or MYBL1‐altered, subtype C [isomorphic]; NET_PLAG1_FUS, neuroepithelial tumour, PLAGL1‐fused; EPN_ST_ZFTA_FUS_C, supratentorial ependymoma, ZFTA fusion‐positive, subclass C. D) t‐SNE highlighting CNS_175 grouped with diffuse glioneuronal tumour, subtype A subgroup. GG, ganglioglioma; PA_CORT, supratentorial pilocytic astrocytoma; GNT_A, diffuse glioneuronal tumour, subtype A; PGNT, papillary glioneuronal tumour. The colour code represents the methylation subclasses within the reference cohort, providing a visual distinction for readers to assess the relative location of a sample within the t‐SNE, as referenced by the DKFZ Brain Tumour Classifier (version 12.8). [file NAN-51-e70041-s007.pdf]

A

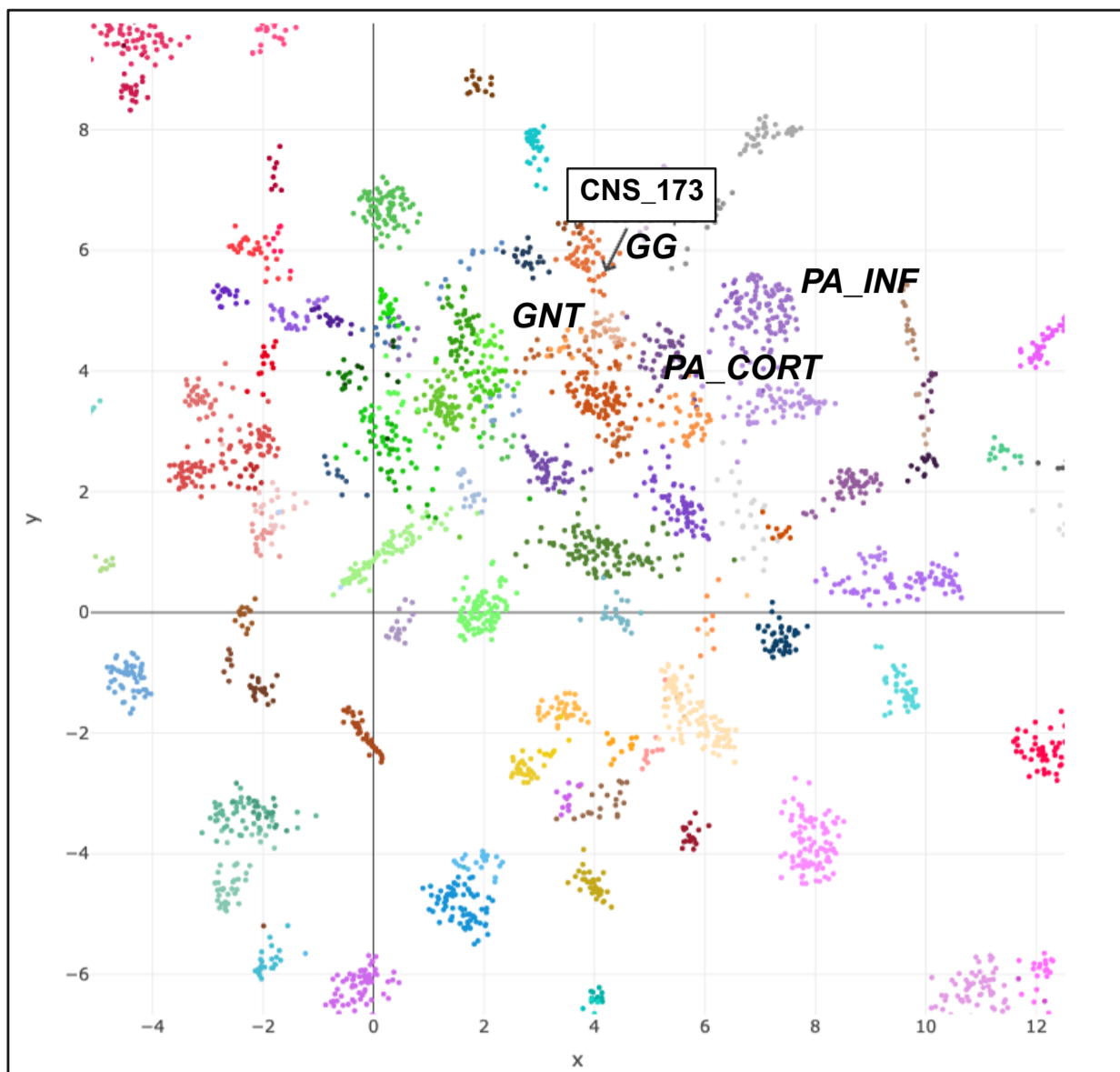

B

**CNS\_102**

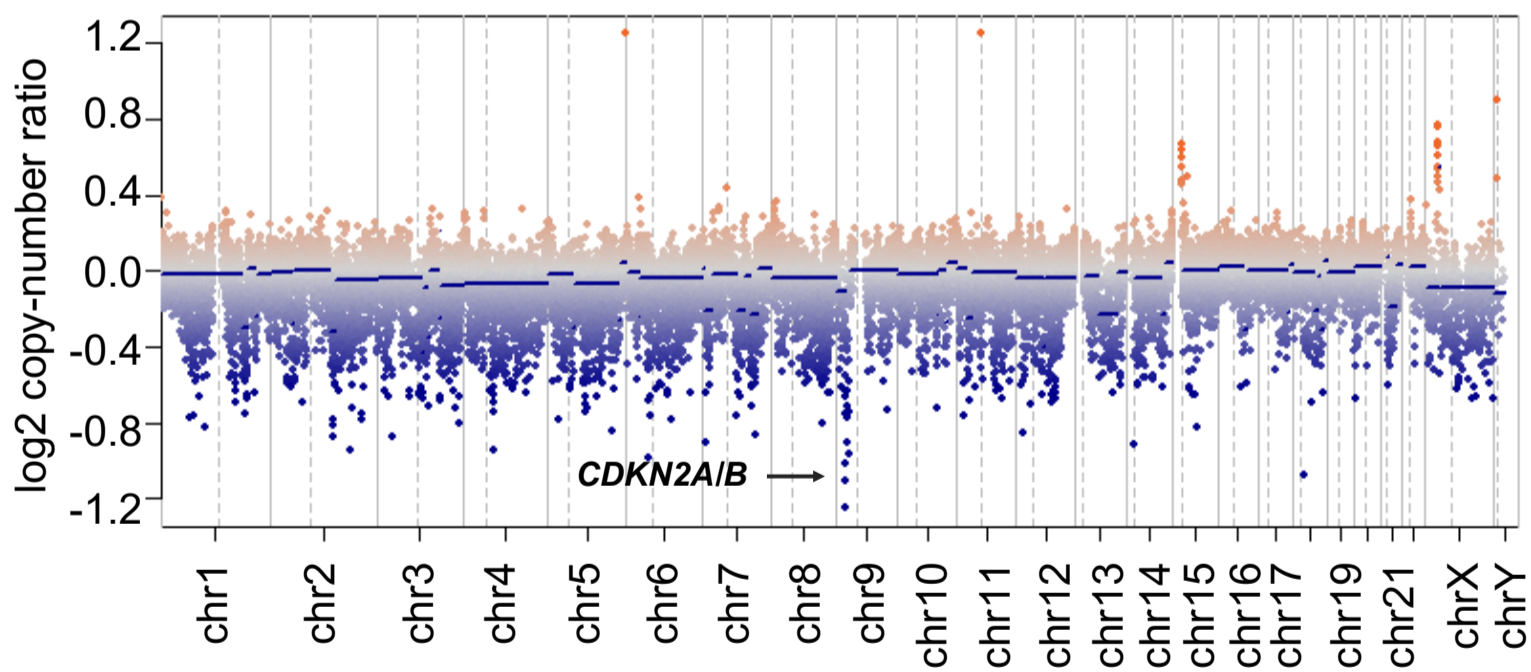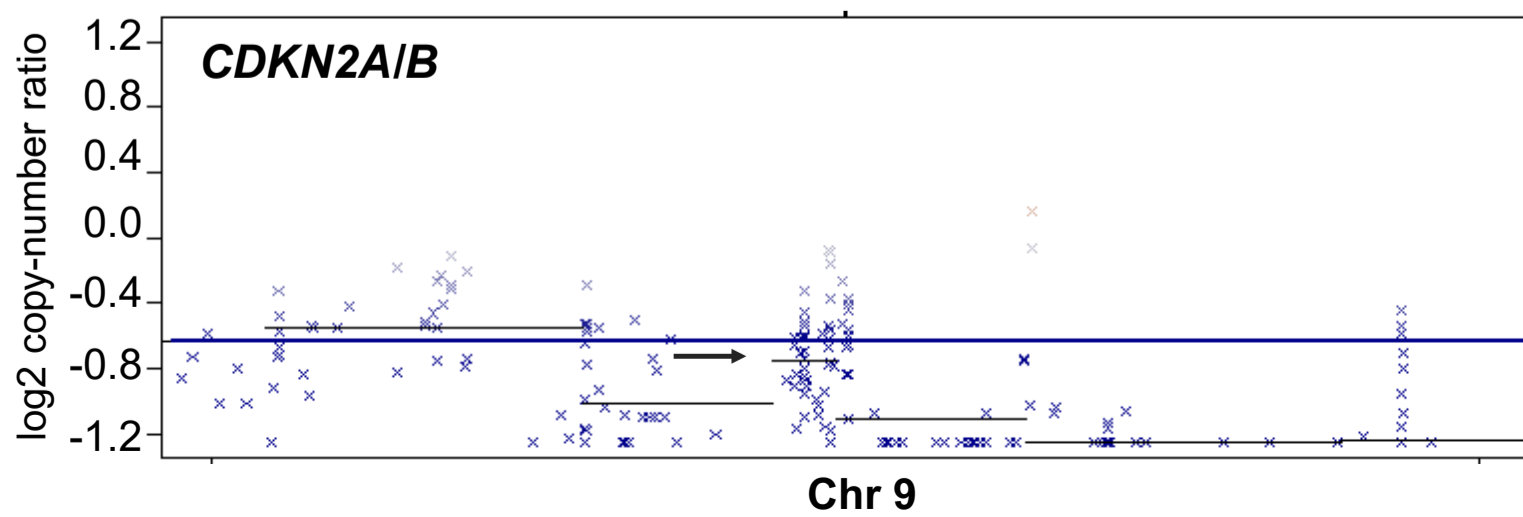

Supplement: Supplementary file 5 — Figure S5: nan70041‐sup‐0005‐figure_S5.pdf. A) t‐SNE highlighting CNS_173 clustered with the ganglioglioma subgroup. GG, ganglioglioma; GNT, diffuse glioneuronal tumour; PA_INF, infratentorial pilocytic astrocytoma; PA_CORT, supratentorial pilocytic astrocytoma. B) Copy number and detailed wrap plots of CNS_102 showing CDKN2A/B deletion. The colour code represents the methylation subclasses within the reference cohort, providing a visual distinction for readers to assess the relative location of a sample within the t‐SNE, as referenced by the DKFZ Brain Tumour Classifier (version 12.8). [file NAN-51-e70041-s002.pdf]
