# Supplementary material for: Use of DNA Methylation Profiling as a Molecular Classification Tool for Paediatric Central Nervous System Tumours: A Middle‐Income Country Population–Based Study
Source: Neuropathol Appl Neurobiol. 2025 Oct 1;51(5):e70041. doi: 10.1111/nan.70041 (PMC12488389; doi:10.1111/nan.70041)
Supplement: Supplementary file 6 — Table S1: Distribution of tumour histology according to the initial diagnosis. [file NAN-51-e70041-s001.docx]

**Supplementary Table 1.** Distribution of tumour histology according to the initial diagnosis**.**

| **Histological group** | **Histological subgroup** | **N** | **%** |
| --- | --- | --- | --- |
| Choroid plexus tumours (n = 9) | Choroid plexus carcinoma | 6 | 10.9 |
|  | Choroid plexus papilloma | 3 | 5.5 |
| Diffuse astrocytic and oligodendroglial  (n =8) | Anaplastic astrocytoma | 1 | 1.8 |
|  | Diffuse astrocytoma | 2 | 3.6 |
|  | Epithelioid glioblastoma | 1 | 1.8 |
|  | Multiform glioblastoma | 2 | 3.6 |
|  | Oligodendroglioma | 1 | 1.8 |
|  | Mixed glioma | 1 | 1.8 |
| Other astrocytic tumours  (n= 51) | Pilocytic astrocytoma | 46 | 83.7 |
|  | Pilomyxoid astrocytoma | 3 | 5.5 |
|  | Pleomorphic xanthoastrocytoma | 2 | 3.6 |
| Embryonal tumour (n = 43) | Atypical rhabdoid teratoid | 3 | 5.5 |
|  | Classic medulloblastoma | 23 | 41.9 |
|  | Desmoplastic medulloblastoma | 8 | 14.6 |
|  | Large-cell medulloblastoma | 1 | 1.8 |
|  | Nodular medulloblastoma | 2 | 3.6 |
|  | CNS embryonal tumour | 6 | 10.9 |
| Ependymomas  (n = 19) | Anaplastic ependymoma | 4 | 7.3 |
|  | Ependymoma | 12 | 21.8 |
|  | Ependymoma RELA positive | 1 | 1.8 |
|  | Subependymoma | 2 | 3.6 |
| Germ cell tumours  (n = 7) | Germinoma | 2 | 3.6 |
|  | Immature teratoma | 3 | 5.5 |
|  | Mature teratoma | 1 | 1.8 |
|  | Mixed germ cell tumour | 1 | 1.8 |
| Meningiomas  (n = 3) | Meningioma | 2 | 3.6 |
|  | Angiomatous Meningioma | 1 | 1.8 |
| Neuronal and mixed neuronal-glial tumours  (n = 22) | Dysembryoplastic neuroepithelial tumour | 8 | 14.6 |
|  | Ganglioglioma | 13 | 23.7 |
|  | Anaplastic ganglioglioma | 1 | 1.8 |
| Tumours of the cranial and paraspinal nerves  (n = 3) | Schwannoma | 3 | 5.5 |
| Tumours of the pineal region (n = 5) | Pineoblastoma | 3 | 5.5 |
|  | Benign pineal cyst | 1 | 1.8 |
|  | Pineocytoma | 1 | 1.8 |
| Tumours of the sellar region  (n = 8) | Craniopharyngioma | 4 | 7.3 |
|  | Adamantinomatous craniopharyngioma | 3 | 5.5 |
|  | Adamantinomatous craniopharyngioma and scamopapilary | 1 | 1.8 |
| Others gliomas  (n = 1) | Angiocentric glioma | 1 | 1.8 |
| Other tumours  (n = 3) | Primary malignant CNS melanoma | 1 | 1.8 |
|  | Intracerebral myxoma | 1 | 1.8 |
|  | Neurocytoma | 1 | 1.8 |
